# Characterization of ferroptosis-triggered pyroptotic signaling in heart failure

**DOI:** 10.1038/s41392-024-01962-6

**Published:** 2024-09-25

**Authors:** Xukun Bi, Xiaotian Wu, Jiaqi Chen, Xiaoting Li, Yangjun Lin, Yingying Yu, Xuexian Fang, Xihao Cheng, Zhaoxian Cai, Tingting Jin, Shuxian Han, Meihui Wang, Peidong Han, Junxia Min, Guosheng Fu, Fudi Wang

**Affiliations:** 1 Key Laboratory of Cardiovascular Intervention and Regenerative Medicine of Zhejiang Province, Department of Cardiology, Sir Run Run Shaw Hospital, Zhejiang University School of Medicine, Hangzhou, China; 2 The Second Affiliated Hospital, School of Public Health, State Key Laboratory of Experimental Hematology, Zhejiang University School of Medicine, Hangzhou, China; 3 The First Affiliated Hospital, Institute of Translational Medicine, Zhejiang University School of Medicine, Hangzhou, China; 4 Department of Nutrition and Toxicology, School of Public Health, Hangzhou Normal University, Hangzhou, China; 5 Center for Genetic Medicine, The Fourth Affiliated Hospital, Zhejiang University School of Medicine, Hangzhou, China; 6 Institute of Genetics, Zhejiang University School of Medicine, Hangzhou, China; 7 School of Public Health, School of Basic Medical Sciences, The First Affiliated Hospital, Hengyang Medical School, University of South China, Hengyang, China; 8 School of Public Health, School of Basic Medical Sciences, The First Affiliated Hospital, Xinxiang Medical University, Xinxiang, China

**Keywords:** Cell biology, Cardiology

## Abstract

Pressure overload–induced cardiac hypertrophy is a common cause of heart failure (HF), and emerging evidence suggests that excessive oxidized lipids have a detrimental effect on cardiomyocytes. However, the key regulator of lipid toxicity in cardiomyocytes during this pathological process remains unknown. Here, we used lipidomics profiling and RNA-seq analysis and found that phosphatidylethanolamines (PEs) and Acsl4 expression are significantly increased in mice with transverse aortic constriction (TAC)–induced HF compared to sham-operated mice. In addition, we found that overexpressing Acsl4 in cardiomyocytes exacerbates pressure overload‒induced cardiac dysfunction via ferroptosis. Notably, both pharmacological inhibition and genetic deletion of Acsl4 significantly reduced left ventricular chamber size and improved cardiac function in mice with TAC-induced HF. Moreover, silencing Acsl4 expression in cultured neonatal rat ventricular myocytes was sufficient to inhibit hypertrophic stimulus‒induced cell growth. Mechanistically, we found that Acsl4-dependent ferroptosis activates the pyroptotic signaling pathway, which leads to increased production of the proinflammatory cytokine IL-1β, and neutralizing IL-1β improved cardiac function in *Acsl4* transgenic mice following TAC. These results indicate that ACSL4 plays an essential role in the heart during pressure overload‒induced cardiac remodeling via ferroptosis-induced pyroptotic signaling. Together, these findings provide compelling evidence that targeting the ACSL4-ferroptosis-pyroptotic signaling cascade may provide a promising therapeutic strategy for preventing heart failure.

## Introduction

Heart failure (HF) is a leading cause of morbidity and mortality worldwide, and its prevalence is projected to increase by 46% from 2012 to 2030, affecting over 8 million people ≥18 years of age by the end of the decade.^1,2^ Hypertrophic growth of the myocardium has been considered a major pathogenic factor in HF. Despite the recent introduction of therapies that target cardiac hypertrophy—including several promising new drugs—the mortality rate remains high, highlighting the urgent need to identify new therapeutic targets and the mechanisms that underlie cardiac hypertrophy.^3^

In the heart, metabolic plasticity is essential for maintaining both a high workload and a variable workload, and a shift in substrate utilization can occur in the heart during hemodynamic stress.^4,5^ The aerobically healthy hearts prefer to use free fatty acids for their primary source of energy supply.^6^ The uptake of exogenous fatty acids to the cardiomyocytes is mediated through specific transporters, including CD36, fatty acid-binding proteins and fatty acid transport proteins, and then be consumed in mitochondria for ATP production. Fatty acid oxidation is an essential step for efficient energy production. In normal hearts, the fatty acid oxidation firstly utilizes fatty acetyl-CoA to generates acetyl-CoA. The acetyl-CoA further enters the tricarboxylic acid cycle to produce NADH and FADH2 which transfer to the electron transport chain to produce ATP for cardiac energy support.^7^ In advanced HF, fatty acid oxidation decreases, accompanied by an increase in glucose utilization.^8,9^ Notably, although fatty acid oxidation is reduced, fatty acid uptake is not reduced. In more advanced stages of HF, fatty acid levels in the plasma are elevated, increasing their delivery to cardiomyocytes.^10^ This process leads to an accumulation of toxic lipid intermediates in the myocardium,^11^ which may act as a signal to ultimately affect cardiac function. Thus, targeting these metabolic intermediates and oxidative stress may slow the development of HF.

The acyl-coenzyme A synthetase long-chain (ACSL) family of proteins are key enzymes in the biosynthesis of fatty acids and phosphatidylethanolamines (PEs) and are essential for fatty acid metabolism and phospholipid remodeling.^12^ ACSLs are key enzymes participating in fatty acid oxidation for esterizing fatty acids to generate fatty acetyl-CoA.^13^ The ACSL family contains 5 members (ACSL1 and ACSL3 through ACSL6), each of which has a specific combination of substrate preference, enzyme kinetics, and response.^14^ ACSL1 is the most intensively studied enzyme in the ACSL family. It is expressed in highly oxidative tissues such as the heart, brown adipose tissue, and skeletal muscle. Cardiac *Acsl1* knockout mice have impaired fatty acid oxidation and exhibit cardiac hypertrophy.^15^ Nevertheless, studies of transgenic *Acsl1* overexpression mice have yielded inconsistent results. For instance, Acsl1 overexpressed in the heart was shown to lead to cardiac lipotoxicity. The mice developed modest systolic dysfunction with mild left ventricular hypertrophy, and it is associated with an increase in reactive oxygen species and fatty acid uptake.^16^ However, impaired cardiac function caused by transverse aortic constriction (TAC) was shown to be further reduced in *Acsl1* transgenic mice by reducing cardiac lipotoxicity.^17^ ACSL4 was recently shown to control sensitivity to ferroptosis by altering cellular lipid composition.^18^ Moreover, the free polyunsaturated fatty acids to form acyl-CoA derivatives is catalyzed by ACSL4, thereby participating in the biosynthesis of cellular membranes. Polyunsaturated fatty acids, including arachidonic acid or linoleic acid, and their elongation products, sensitize cells to undergo oxidation.^19^ This type of oxidation involves a reaction replacing a hydrogen atom with a peroxyl group, as a result, only specific lipids are susceptible to peroxidation that is dependent on the strength of carbon-hydrogen bonds.^20^ In addition, an accumulation of lipid peroxidation causes damage to cellular membranes which driving ferroptosis.^21,22^ Arachidonic acid-pretreated cells are sensitized to ferroptosis, whereas cells supplemented with polyunsaturated fatty acid replaced with deuterium atoms at the site of peroxidation could block ferroptosis.^23^ In addition, monounsaturated fatty acids, such as oleic acid, could increase the resistance to ferroptosis when these lipids are incorporated into membrane lipids.^24^ ACSL4 is also an essential component for ferroptosis execution.^25^ Loss of Acsl4 decreases the enrichment of long polyunsaturated fatty acid in cell membranes and therefore shows the decline of substrates for lipid peroxidation and exhibits ferroptosis resistance.^18,26^ Even though ACSL family has been intensively studied in fatty acid metabolism and ferroptosis, the role of ACSL family members in the heart in both health and disease remains contradictory and therefore needs to be explored.

Given the important role that ACSL family members play in lipid metabolism, our goal was to identify which ACSL isoform contributes to HF using a murine model in which pressure overload-induced HF is induced by TAC. Screening mice with TAC-induced HF revealed that Acsl4 was significantly upregulated compared to sham-operated mice. Furthermore, we found that overexpressing Acsl4 selectively in cardiomyocytes promotes cardiac hypertrophy and the progression of HF by activating ferroptosis-induced pyroptotic signaling. These findings indicate that ACSL4, ferroptosis, and pyroptosis play essential roles in promoting HF under pressure overload and may represent novel therapeutic targets.

## Results

### Screening TAC-induced cardiac hypertrophy reveals significantly increased phosphatidylethanolamines (PEs) and upregulated Acsl4 expression

**Fig. 1:**
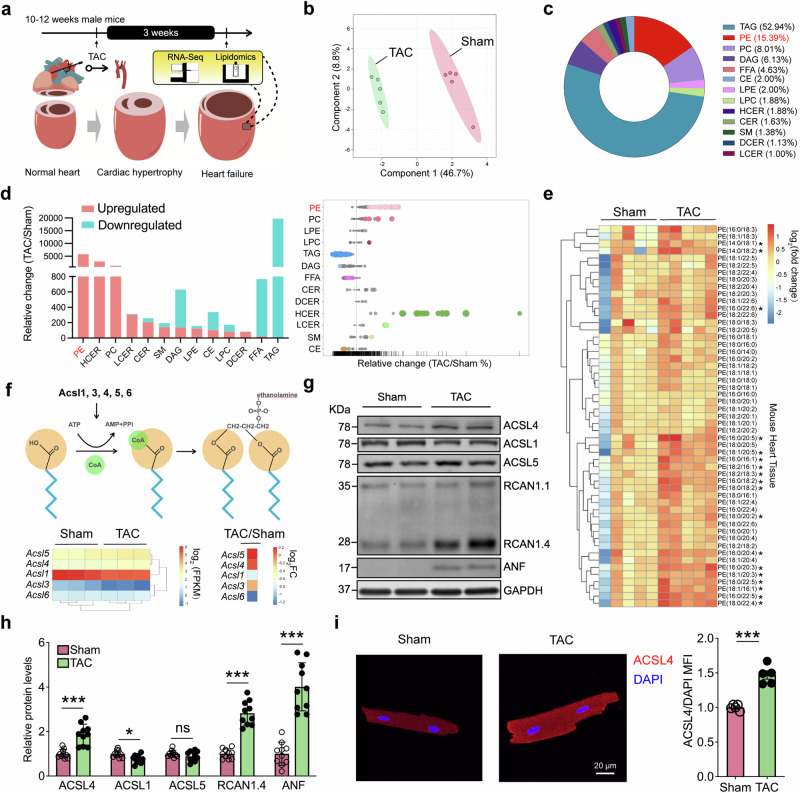
TAC-induced cardiac hypertrophy leads to an upregulation of Acsl4. **a** Diagram depicting the strategy for lipidomics profiling and RNA-seq analysis 3 weeks after performing transverse aortic constriction (TAC) or sham surgery. Also depicted is cardiac outcome, with TAC-induced cardiac hypertrophy progressing to heart failure (Created with BioRender.com). **b** Partial least squares discriminant analysis (PLS-DA) scores plotted for the lipidomics data for left ventricular tissue in sham-operated and TAC-operated mice (*n* = 5 mice/group). **c** Distribution of the indicated classes of metabolites detected in cardiac tissues using targeted lipidomics profiling. **d** Summary of the relative change in the levels of the indicated lipid classes measured in TAC-operated mice relative to sham-operated mice; upregulated and downregulated lipid species are indicated in red and blue, respectively. Note the break in the *y*-axis. **e** Heatmap summarizing the fold change in the relative abundance of the indicated phosphatidylethanolamines (PEs) metabolites in sham-operated and TAC-operated mice (*n* = 5 mice/group). **f** Schematic diagram (top) depicting the metabolic pathways mediated by ACSL family members, and heatmaps of fragments per kilobase per million mapped fragments (FPKM, bottom left) and log_2_ fold change (FC, bottom right) for the indicated *Acsl* genes based on the RNA-seq data. Western blot analysis (**g**) and quantification (**h**) of the indicated proteins in the heart tissue of sham-operated and TAC-operated mice (*n* = 10 mice/group). **i** Sections of adult mouse ventricular cardiomyocytes obtained from sham-operated and TAC-operated mice were immunostained for ACSL4 (red); the nuclei were counterstained with DAPI (blue). Scale bar, 20 μm. Shown at the right is the mean fluorescence intensity (MFI) of ACSL4 immunostaining normalized to DAPI intensity measured in the indicated groups (*n* = 5 mice/group). In this and subsequent figures, unless indicated otherwise summary data are presented as the mean ± SEM. *P < 0.05, ***P < 0.001, and ns, not significant (unpaired Student’s *t*-test)

Metabolic remodeling is important in the pathophysiology of HF. Because a healthy heart relies primarily on fatty acid oxidation to produce ATP as fuel,^5^ we first looked for changes in lipid metabolism in adult wild-type (WT) mice following TAC to induce pressure overload. After 3 weeks, the mice in the TAC group first developed cardiac hypertrophy, which later progressed to HF; specifically, compared to sham-operated mice, the TAC-operated mice had reduced cardiac contractile function and left ventricular hypertrophy (Supplementary Fig. 1a–f). In addition, we measured a pressure gradient of ~40 mmHg in the TAC-operated mice (Supplementary Fig. 1g, h). As an additional control, we found no difference in TAC-induced cardiac outcome between WT mice and Myh6-Cre mice, which express Cre recombinase driven by the *Myh6* promoter are were subsequently used to target expression in cardiomyocytes (Supplementary Fig. 1). Three weeks after surgery, we also performed lipidomics and RNA sequencing (RNA-seq) analyses on left ventricular tissues (Fig. 1a). Partial least squares discriminant analysis of the lipidomics data clearly distinguished between the mice in the TAC group and the sham-operated mice (Fig. 1b). Moreover, our lipidomics analysis revealed a total of 799 lipids in 13 distinct lipid classes (Fig. 1c), with phosphatidylethanolamines (PEs) significantly increased in the TAC group compared to the sham group (Fig. 1d). Further analysis revealed that specific PEs such as PE (18:0/22:4), PE (16:0/22:5), and PE (16:0/20:4) were among the top increased PEs in the TAC-treated hearts (Fig. 1e). Next, we analyzed a previously published set of human metabolomics data from patients with hypertrophic cardiomyopathy (HCM).^27^ Among the 18 PE species we identified as increased in the mice with HF, 6 were also found to be increased in patients with HCM compared to control subjects (Supplementary Fig. 2). We also compared the expression level of *ACSL4* between 13 patients with HCM who underwent septal myectomy and 7 donor subjects with no major cardiac history^28^ and found slightly—albeit not significantly—higher expression among the patients with HCM compared to controls (Supplementary Fig. 2).

The first step in the biosynthesis of PEs is the ACSL-mediated thioesterification of fatty acids to produce long-chain acyl-CoA.^29^ To examine whether ACSL family members play a pathogenic role in HF, we measured the transcripts of all five *Acsl* genes in the cardiac RNA-seq data analyzed 3 weeks after TAC/sham surgery. As shown in Fig. 1f, and confirmed using RT-qPCR (Supplementary Fig. 3a), both *Acsl4* and *Acsl5* mRNA levels were higher in the TAC group compared to the sham group; however, at the protein level only Acsl4 was higher in the TAC group (Fig. 1g, h). We then measured ACSL4 proteins in left ventricular tissue using immunostaining. As shown in Fig. 1i, Acsl4 levels were higher in the TAC group compared to the sham group; this increase was significant both 7 and 21 days after TAC (Supplementary Fig. 3). These results indicate that Acsl4 expression is increased in TAC-induced cardiac hypertrophy and may therefore play a role in disease progression.

### Overexpressing Acsl4 in cardiomyocytes aggravates heart failure in mice

**Fig. 2:**
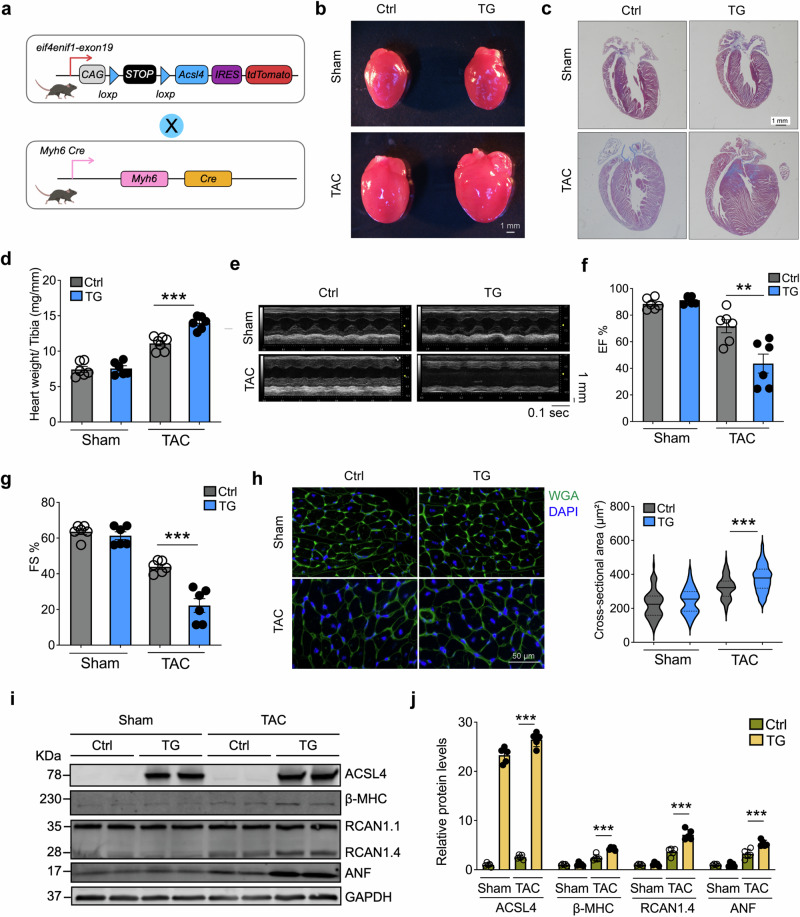
Cardiomyocyte-specific *Acsl4* transgenic mice have increased cardiac pathology in response to TAC-induced pressure overload. **a** Strategy for generating *Acsl4* TG mice overexpressing Acsl4 selectively in cardiomyocytes. Myh6-Cre and/or loxP-stop-loxP-Acsl4 mice were used as a control (Ctrl) group. **b** Images of whole hearts obtained from sham-operated and TAC-operated Ctrl and TG mice. Scale bar, 1 mm. **c** Heart sections were prepared from sham-operated and TAC-operated Ctrl and TG mice and stained with Masson’s trichrome to measure cardiac fibrosis; scale bar, 1 mm. **d** Summary of the heart weight/tibia length ratio measured in sham-operated and TAC-operated Ctrl and TG mice (*n* = 6 mice/group). M-mode echocardiography images (**e**) and summary of ejection fraction (**f**; *n* = 6 mice/group) and fractional shortening (**g**; *n* = 6 mice/group) measured in the indicated mice. **h** Heart sections were prepared from the indicated mice and stained with wheat germ agglutinin (WGA, green) in order to calculate cardiomyocyte cross-sectional area; the nuclei were counterstained with DAPI (blue). Scale bar, 50 μm. Shown at the right is a violin plot summarizing the relative cardiac cell cross-sectional area measured in the indicated mice (*n* = 81–92 cardiomyocytes from 6 mice/group). Western blot analysis (**i**) and quantification (**j**) of ACSL4 and the cardiac hypertrophy markers β-MHC (β-myosin heavy chain), RCAN1.4, and ANF (atrial natriuretic factor) measured in hearts isolated from sham-operated and TAC-operated Ctrl and TG mice (*n* = 5 mice/group). **P < 0.01 and ***P < 0.001 (two-way ANOVA followed by Tukey’s multiple comparisons test)

To investigate the putative role of increased Acsl4 expression in cardiac hypertrophy, we generated an *Acsl4* transgenic (*Acsl4* TG) mouse in which expression of the *Acsl4* transgene is activated by cardiomyocyte-specific Cre (Myh6-Cre),^30^ thereby overexpressing Acsl4 selectively in cardiomyocytes (Fig. 2a); for these experiments, Myh6-Cre and/or loxP-stop-loxP-Acsl4 mice were used as negative controls. We found no significant difference between *Acsl4* TG and control mice with respect to heart size, shape, or fibrosis (Fig. 2b–d); in addition, echocardiography and histology showed no difference in heart rate, systolic function, chamber size, or posterior wall thickness between *Acsl4* TG and control mice (Supplementary Fig. 4). These results indicate that simply overexpressing Acsl4 in the heart is not sufficient to affect cardiac structure or function.

We then asked whether overexpressing Acsl4 affected the progression of cardiac hypertrophy and altered cardiac function after pressure overload (i.e., TAC) by examining control and *Acsl4* TG mice 3 weeks after sham or TAC surgery. Consistent with our findings in WT mice, compared to sham-operated control mice, the TAC-operated control mice developed a clear increase in heart mass reflected by an increase in the heart weight/tibia length ratio, and this increase was even larger in TAC-operated *Acsl4* TG mice (Fig. 2b–d). We also found that cardiac contractility was decreased in TAC-operated controls compared to sham-operated controls and was decreased even further in TAC-operated *Acsl4* TG mice based on reduced ejection fraction and fractional shortening (Fig. 2e–g). Consistent with these findings, wheat germ agglutinin (WGA) staining of heart sections showed that the cross-sectional area of cardiomyocytes was significantly increased in the TAC-operated *Acsl4* TG mice compared to TAC-operated controls (Fig. 2h); moreover, several markers of cardiac hypertrophy, including β-MHC, RCAN1.4, and ANF, were significantly increased in the TAC-operated *Acsl4* TG mice compared to TAC-operated controls (Fig. 2i, j). We also confirmed that Acsl4 is overexpressed in the heart tissue of *Acsl4* TG mice at both the mRNA (Supplementary Fig. 4i) and protein (Fig. 2i) levels. Taken together, these findings suggest that overexpressing Acsl4 in cardiomyocytes exacerbates cardiac remodeling and dysfunction induced by pressure overload.

### Loss of Acsl4 in cardiomyocytes reduces TAC-induced pathological remodeling and cardiac dysfunction

**Fig. 3:**
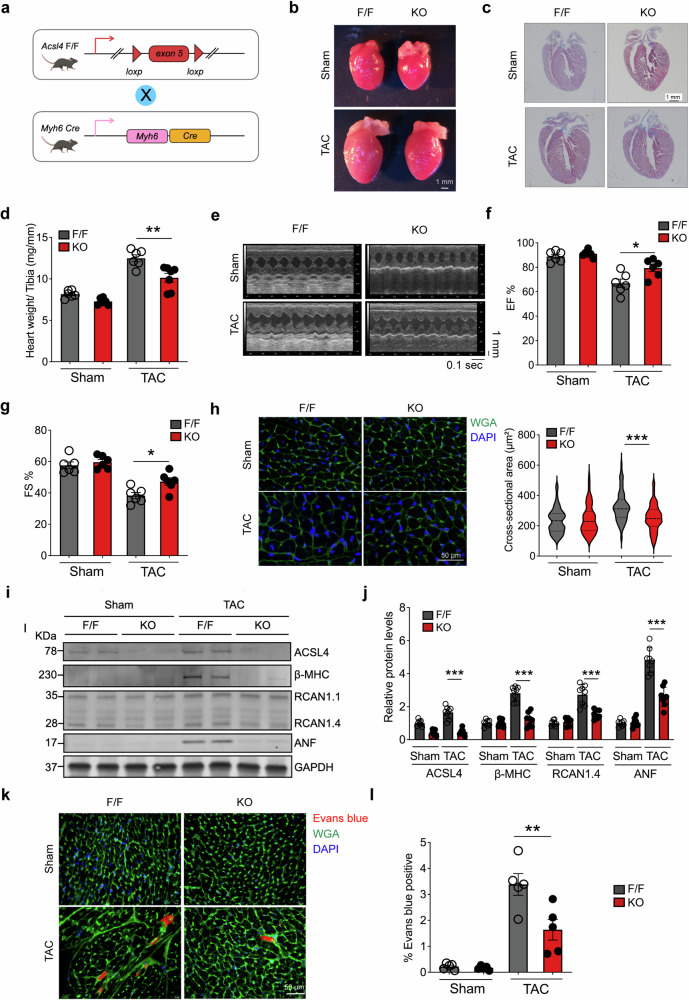
Pharmacologically inhibiting Acsl4 and knocking out Acsl4 selectively in cardiomyocytes protect against TAC-induced heart failure. **a** Strategy for generating cardiomyocyte-specific *Acsl4* knockout (*Acsl4* KO) mice. *Acsl4*^*flox/flox*^ (*Acsl4* F/F) mice were used as the control group. **b** Images of whole hearts obtained from sham-operated and TAC-operated *Acsl4* F/F and *Acsl4* KO mice; scale bar, 1 mm. **c** Heart sections were prepared from sham-operated and TAC-operated *Acsl4* F/F and *Acsl4* KO mice and stained with Masson’s trichrome; scale bar, 1 mm. **d** Summary of the heart weight/tibia length ratio in sham-operated and TAC-operated *Acsl4* F/F and *Acsl4* KO mice (*n* = 6 mice/group). M-mode echocardiography images (**e**) and summary of ejection fraction (**f**; *n* = 6 mice/group) and fractional shortening (**g**; *n* = 6 mice/group) measured in the indicated mice. **h** Heart sections were prepared from the indicated mice and stained with WGA (green) to calculate cardiomyocyte cross-sectional area; the nuclei were counterstained with DAPI (blue). Scale bar, 50 μm. Shown at the right is a violin plot summarizing the relative cardiac cell cross-sectional area measured in the indicated mice (*n* = 85–112 cardiomyocytes from 6 mice/group). Western blot analysis (**i**) and quantification (**j**) of ACSL4, β-MHC, RCAN1.4, and ANF measured in hearts isolated from the indicated mice (*n* = 8 mice/group). **k** Sham-operated and TAC-operated *Acsl4* F/F and *Acsl4* KO mice received an i.p. injection of Evans blue dye, and heart sections were subsequently prepared and stained with WGA (green); Evans blue‒positive cardiomyocytes are indicated in red, and the nuclei were counterstained with DAPI (blue). Scale bar, 50 μm. Summary of Evans blue‒positive cells in heart sections obtained from the indicated mice treated as shown in (**l**); (*n* = 5 mice/group). *P < 0.05, **P < 0.01, and ***P < 0.001 (two-way ANOVA followed by Tukey’s multiple comparisons test)

Given the severe cardiac phenotype observed in *Acsl4* TG mice following TAC, we asked whether cardiac Acsl4 is necessary for pathological remodeling and/or the progression of HF in response to pressure overload. We therefore generated cardiac-specific *Acsl4* knockout (*Acsl4* KO) mice (Fig. 3a) by crossing *Acsl4*^*flox/flox*^ conditional knockout (*Acsl4* F/F) mice with Myh6-Cre mice.

Similar to the *Acsl4* TG mice, we found no clear change in cardiac structure or function in *Acsl4* KO mice compared to *Acsl4* F/F control mice (Fig. 3b–d and Supplementary Fig. 5). Thus, we conclude that loss of Acsl4 in cardiomyocytes is not sufficient to affect cardiac development or function.

When subjected to hemodynamic stress, however, TAC-induced increase in heart mass (Fig. 3d), reduced systolic function (Fig. 3e–g), and enlarged cross-sectional area of ventricular cardiomyocytes (Fig. 3h) were all significantly lower in the *Acsl4* KO mice compared to TAC-operated *Acsl4* F/F mice. In addition, TAC increased the levels of all three markers of cardiac hypertrophy (namely, β-MHC, RCAN1.4, and ANF) in the *Acsl4* F/F mice, and this increase was virtually eliminated in TAC-operated *Acsl4* KO mice (Fig. 3i, j). As expected, cardiac Acsl4 expression was drastically reduced in *Acsl4* KO mice at the protein levels (Fig. 3i, j) and mRNA (Supplementary Fig. 5j) levels; the residual Acsl4 protein and mRNA present in the *Acsl4* KO mice is likely due to Acsl4 expressed in heart cells other than cardiomyocytes.

To detect cell death, we injected Evans blue intraperitoneally and measured its presence in cardiomyocytes after inducing TAC. In WT mice, with the progression from cardiac hypertrophy to HF (Supplementary Fig. 6a–h), we found that ~15% of cardiomyocytes were positive for Evans blue 3 days after TAC; although this percentage decreased to only 3% by 21 days post-TAC, this was still significantly higher than in sham-operated controls, in which virtually no Evans blue–positive cells were detected (Supplementary Fig. 6i, j). Next, we measured Evans blue in TAC-operated *Acsl4* KO mice and found significantly fewer Evans blue‒positive cardiomyocytes compared to TAC-operated *Acsl4* F/F mice (Fig. 3k, l).

These findings suggest that loss of Acsl4 in cardiomyocytes reduces pathological remodeling induced by hemodynamic stress. Consistent with this notion, we also found that treating TAC-operated WT mice with PRGL493 (Supplementary Fig. 7) and pioglitazone (Supplementary Fig. 8), two potent and selective inhibitors of Acsl4, partially protected against cardiac hypertrophy, reduced the expression of cardiac hypertrophy markers, and improved ventricular function.

### ACSL4 is required for the hypertrophic growth of cardiomyocytes in vitro

**Fig. 4:**
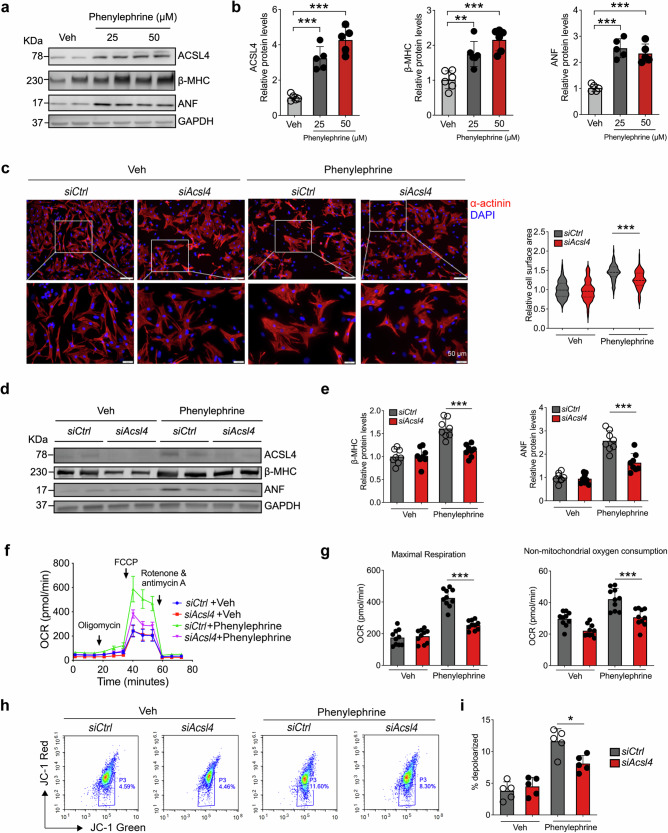
Acsl4 is required for the hypertrophic growth of neonatal rat ventricular myocytes. Western blot analysis (**a**) and quantification (**b**) of the protein levels of ACSL4 and the hypertrophy markers β-MHC and ANF measured in cultured neonatal rat ventricular myocytes (NRVMs) treated with vehicle, 25 μM phenylephrine, or 50 μM phenylephrine for 24 h (*n* = 5 biological replicates/group). **c** Representative fluorescence images of NRVMs immunostained for α-actinin; where indicated, the cells were transfected with a control siRNA (*siCtrl*) or an *Acsl4*-targeted siRNA (*siAcsl4*) after 24 h in the presence of vehicle or 50 μM phenylephrine; scale bars, 50 μm. Shown at the right is a violin plot summarizing the relative cross-sectional area of NRVMs treated as indicated (n = 90–94 cardiomyocytes quantified from 5 biological replicates/group). Western blot analysis (**d**) and quantification (**e**) of ACSL4, β-MHC, and ANF measured in NRVMs treated as indicated (*n* = 8 biological replicates/group). Time course of oxygen consumption rate (**f**) and summary of maximal respiration (**g**, left) and non-mitochondrial oxygen consumption rate (**g**, right) measured using Seahorse analysis in NRVMs treated as indicated (*n* = 10 biological replicates/group). Flow cytometry analysis using JC-1 to measure mitochondrial membrane depolarization (**h**) and summary of the percentage of cells with depolarized mitochondrial membranes (**i**) in the indicated groups (*n* = 5 biological replicates/group). *P < 0.05, **P < 0.01, and ***P < 0.001 (one-way or two-way ANOVA followed by Tukey’s multiple comparisons test)

The adrenergic alpha1 receptor agonist phenylephrine is most commonly applied to hypertrophic growth stimulation of cardiomyocytes in vitro.^31,32^ To determine whether the expression of Acsl4 is altered in cardiomyocytes in response to hypertrophic stimuli, we isolated and cultured neonatal rat ventricular myocytes (NRVMs) from 1-3-day-old Sprague-Dawley rat pups and then treated the cells with phenylephrine for 24 h. We found that phenylephrine treatment caused an increase in the hypertrophic markers β-MHC and ANF (Fig. 4a, b). Importantly, phenylephrine treatment also significantly increased ACSL4 protein levels (Fig. 4a, b), reminiscent of the in vivo findings in TAC-operated mice. We then asked whether knocking down *Acsl4* expression in NRVMs using siRNA (*siAcsl4*) can prevent phenylephrine-induced hypertrophic growth. We found that phenylephrine increased relative cell surface area (i.e., caused by cellular hypertrophy) measured using α-actinin immunostaining, and this increase was significantly reduced in *siAcsl4*-treated cells, but not in cells treated with a control siRNA (*siCtrl*) (Fig. 4c). Moreover, the phenylephrine-induced increases in hypertrophic markers and Acsl4 were significantly reduced in *siAcsl4*-treated NRVMs (Fig. 4d, e).

We then measured oxygen consumption rate in NRVMs using Seahorse analysis and found that phenylephrine treatment increased maximal respiration and non-mitochondrial oxygen consumption, and these increases were significantly reduced in *siAcsl4*-treated cells (Fig. 4f, g). We also used the dye JC-1 to measure mitochondrial membrane potential in NRVMs, with reduced red fluorescence indicating membrane depolarization and found that phenylephrine caused an increase in mitochondrial depolarization, an effect that was significantly reduced in *siAcsl4*-treated cells (Fig. 4h, i). Together, these findings suggest that knocking down Acsl4 in cardiomyocytes at least partially protects against phenylephrine-induced cellular hypertrophy and mitochondrial dysfunction.

To investigate the effects of phenylephrine on the integrity of the plasma membrane in cardiomyocytes, we examined NRVMs using transmission electron microscope and we found phenylephrine increased NRVMs death stained by propidium iodide (PI) by flow cytometric analysis, and this effect was reduced significantly in *siAcsl4*-treated cells (Supplementary Fig. 9a). Similarly, we found an increase in ruptured membranes in phenylephrine-treated cells, and this effect was reduced considerably in *siAcsl4*-treated cells (Supplementary Fig. 9b). In contrast, we found that overexpressing Acsl4 in NRVMs using an adenovirus (Ad-*Acsl4*) significantly increased the effects of phenylephrine on plasma membrane integrity and PI staining (Supplementary Fig. 9c, d).

### ACSL4-dependent ferroptosis increases the hypertrophic response

**Fig. 5:**
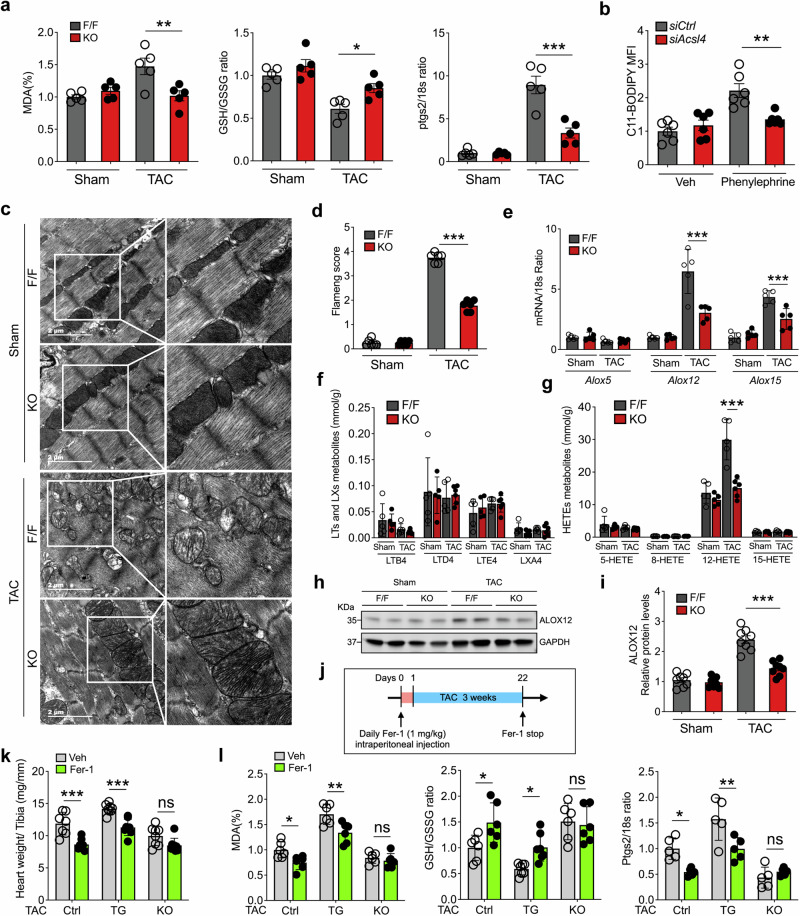
Acsl4-dependent ferroptosis exacerbates TAC-induced cardiac hypertrophy. **a** Summary of the relative change in the ferroptosis markers MDA (malondialdehyde), the GSH/GSSG (reduced glutathione/oxidized glutathione) ratio, and *Ptgs2* (prostaglandin-endoperoxide synthase 2) mRNA measured in sham-operated and TAC-operated *Acsl4* F/F and *Acsl4* KO mice, normalized to the sham-operated *Acsl4* F/F group (*n* = 5 mice/group). **b** Flow cytometry analysis using C11-BODIPY to measure lipid peroxidation in vehicle- or phenylephrine-treated NRVMs transfected with *siCtrl* or *siAcsl4*. Shown is the summary of C11-BODIPY mean fluorescence intensity (MFI) measured in the indicated cells (*n* = 6 biological replicates/group). Representative transmission electron microscopy images (**c**) and corresponding relative mitochondrial Flameng scores (**d**) measured in left ventricular tissues obtained from sham-operated and TAC-operated *Acsl4* F/F and *Acsl4* KO mice; scale bars, 2 μm. **e** Summary of *Alox5*, *Alox12*, and *Alox15* mRNA measured in sham-operated and TAC-operated *Acsl4* F/F and *Acsl4* KO mice, normalized to *18S* rRNA (*n* = 5 mice/group). Summary of the indicated oxylipins (**f**) and HETEs (**g**) measured in heart tissues isolated from sham-operated and TAC-operated *Acsl4* F/F and *Acsl4* KO mice (*n* = 5–6 mice/group). Western blot analysis (**h**) and quantification (**i**) of ALOX12 protein measured in heart tissues isolated from sham-operated and TAC-operated *Acsl4* F/F and *Acsl4* KO mice (*n* = 8 mice/group). **j** Diagram depicting the strategy for treating mice with daily injections of the ferroptosis inhibitor ferrostatin-1 (Fer-1, 1 mg/kg/day) or vehicle one day before and for three weeks after TAC surgery. **k** Summary of the heart weight/tibia length ratio measured in vehicle-treated and Fer-1‒treated TAC-operated control, *Acsl4* TG, and *Acsl4* KO mice (*n* = 7–8 mice/group). **l** Summary of the relative change in the ferroptosis markers MDA, the GSH/GSSG ratio, and *Ptgs2* mRNA measured in vehicle-treated and Fer-1‒treated TAC-operated control, *Acsl4* TG, and *Acsl4* KO mice, normalized to the respective vehicle-treated TAC-operated control group (*n* = 5–6 mice/group). *P < 0.05, **P < 0.01, ***P < 0.001, and ns, not significant (two-way ANOVA followed by Tukey’s multiple comparisons test)

PEs are key phospholipids and may drive cellular ferroptosis in response to oxidative stress. Given that ACSL4 functions as an isozyme in the biosynthesis of PEs, we asked whether ferroptosis participates in pressure overload-induced cardiac remodeling. To address this question, we measured several markers of ferroptosis, including malondialdehyde (MDA), the glutathione/oxidized glutathione ratio (GSH/GSSG), and *Ptgs2* mRNA. We found that all three markers differed significantly between TAC-operated *Acsl4* KO mice and TAC-operated *Acsl4* F/F mice (Fig. 5a), suggesting that the loss of Acsl4 in cardiomyocytes reduces ferroptosis.

Next, we used the fluorescent probe C11-BODIPY to measure lipid peroxidation in NRVMs and found that the phenylephrine-induced increase in C11-BODIPY staining was significantly reduced in *siAcsl4*-treated NRVMs compared to *siCtrl*-treated NRVMs (Fig. 5b). We previously reported that lipid peroxidation in the mitochondria plays a role in ferroptosis;^33^ we therefore examined mitochondrial morphology in the heart tissues of sham-operated and TAC-operated *Acsl4* F/F and *Acsl4* KO mice. We found severely disrupted mitochondria in TAC-operated *Acsl4* F/F mice, but not in TAC-operated *Acsl4* KO mice (Fig. 5c). To quantify this mitochondrial damage, we used the Flameng mitochondrial injury score^33–35^ based on mitochondrial ultrastructure and found reduced Flameng scores (indicating less severe mitochondrial damage) in the cardiac tissue of TAC-operated *Acsl4* KO mice compared to TAC-operated *Acsl4* F/F mice (Fig. 5d), suggesting that the loss of Acsl4 protects against TAC-induced mitochondrial dysfunction.

Studies have reported that the oxidation of polyunsaturated fatty acids in PEs is mediated by the arachidonate lipoxygenase (ALOX) family of enzymes, generating the precursors of bioactive oxylipins^30^ via ferroptotic peroxidation.^36^ We therefore measured *Alox5*, *Alox12*, and *Alox15* mRNA levels using RT-qPCR, and we measured the levels of these enzymes’ downstream oxylipins by liquid chromatography-tandem mass spectrometry. We found that TAC-operated *Acsl4* KO mice had significantly lower levels of *Alox12* and *Alox15* mRNA compared to TAC-operated *Acsl4* F/F mice (Fig. 5e). Consistent with this finding, we also found that TAC-operated *Acsl4* KO mice had significantly lower levels of 12-HETE (12-hydroxyeicosatetraenoic acid, a downstream metabolite of Alox12 activity^37^), with no change in any other downstream metabolites measured (Fig. 5f, g). Moreover, we found that ALOX12 protein levels were higher in TAC-operated *Acsl4* F/F mice compared to sham-operated *Acsl4* F/F mice, but were significantly lower in TAC-operated *Acsl4* KO mice compared to TAC-operated *Acsl4* F/F mice (Fig. 5h, i).

Given that ferroptosis is characterized by an accumulation of ROS in membrane lipids due to reduced GPX4 (glutathione peroxidase 4) activity, we also examined whether phenylephrine alters ROS in *siAcsl4*-treated NRVMs. Using the probe H_2_DCFDA to measure ROS levels, we found that phenylephrine treatment increased ROS levels in NRVMs, and this increase was reduced in *siAcsl4*-treated cells (Supplementary Fig. 10a, b). Similarly, phenylephrine increased ALOX12 protein levels, and this increase was significantly reduced in *siAcsl4*-treated cells (Supplementary Fig. 10c, d). To confirm that oxidative stress increases ALOX12 expression, we applied H_2_O_2_ to the cells in order to induce oxidative stress and found that ALOX12 protein levels were increased in NRVMs in a concentration-dependent manner (Supplementary Fig. 10e, f). ROS scavenger *N*-acetyl-L-cysteine (NAC) significantly reduced ALOX12 protein levels in phenylephrine-treated NRVMs overexpressing *Acsl4* (Supplementary Fig. 10g, h). Together, these results indicate that ROS play a role in the ACSL4-mediated upregulation of ALOX12.

Given our finding that cardiomyocytes lacking Acsl4 have reduced TAC-induced lipid peroxidation, we next asked whether inhibiting lipid peroxidation affects the progression of cardiac hypertrophy in *Acsl4* TG mice. We, therefore, treated control, *Acsl4* TG, and *Acsl4* KO mice with the lipophilic antioxidant and ferroptosis-specific inhibitor Ferrostatin-1 (Fer-1) starting one day before TAC surgery and then continued treating the mice with Fer-1 daily for 3 weeks following TAC (Fig. 5j); as a negative control, we found that Fer-1 treatment had no significant effect on cardiac outcome in sham-operated control, *Acsl4* TG, or *Acsl4* KO mice. In contrast, following TAC heart mass was significantly lower in the Fer-1‒treated control mice compared to vehicle-treated control mice (Fig. 5k). Similarly, heart mass was significantly lower in the Fer-1‒treated *Acsl4* TG mice compared to vehicle-treated *Acsl4* TG mice (Fig. 5k); moreover, Fer-1 decreased MDA levels, increased the GSH/GSSG ratio, and lowered cardiac *Ptgs2* mRNA levels in both control and *Acsl4* TG mice compared to their respective vehicle-treated groups (Fig. 5l). Importantly, however, Fer-1 treatment had no effect on heart mass, MDA levels, the GSH/GSSG ratio, or cardiac *Ptgs2* mRNA levels in TAC-operated *Acsl4* KO mice compared to vehicle-treated *Acsl4* KO mice (Fig. 5k, l). Thus, both ferroptosis and lipid peroxidation appear to play an essential role in TAC-induced cardiac hypertrophy via ACSL4.

### ACSL4-induced ferroptosis triggers the pyroptotic signaling pathway in cardiac hypertrophy

**Fig. 6:**
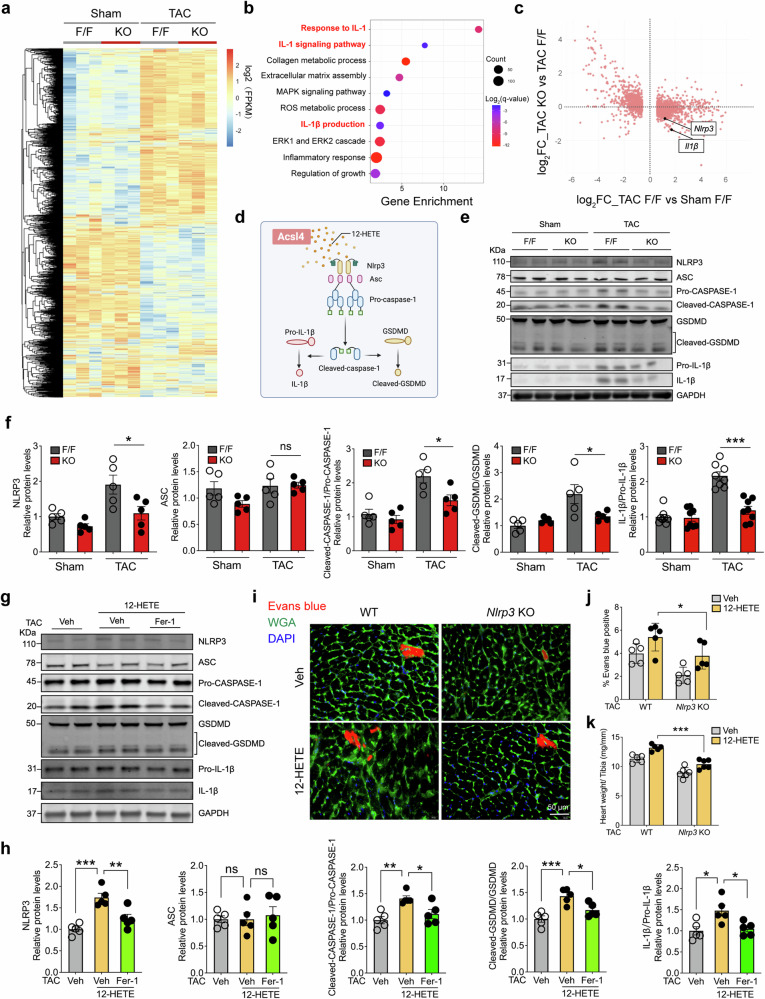
The pyroptotic pathway is activated following Acsl4-induced ferroptosis. **a** Heatmap summarizing the differentially expressed genes in left ventricular tissues obtained from sham-operated and TAC-operated *Acsl4* F/F and *Acsl4* KO mice (*n* = 3 mice/group). **b** KEGG analysis of RNA-seq data showing the top 10 enriched pathways in cardiac tissues obtained from TAC-operated *Acsl4* F/F and *Acsl4* KO mice. **c** Plot of the log_2_ fold change in TAC-operated *Acsl4* KO mice versus TAC-operated *Acsl4* F/F mice (on the *y*-axis) plotted against the log_2_ fold change in TAC-operated *Acsl4* F/F mice versus sham-operated *Acsl4* F/F mice (on the *x*-axis). Both *Il1b* and *Nlrp3* were upregulated in the TAC-operated *Acsl4* F/F mice (i.e., to the right of the *y*-axis), but downregulated in the TAC-operated *Acsl4* KO mice (i.e., below the *x*-axis). **d** Schematic diagram depicting activation of the pyroptotic signaling pathway by 12-HETE, leading to the production of mature IL-1β from pro-IL-1β and the production of cleaved GSDMD from GSDMD (Gasdermin D). Western blot analysis (**e**) and quantification (**f**) of the pyroptotic signaling markers NLRP3 (NLR family pyrin domain containing 3), ASC (apoptosis-associated speck-like protein containing a CARD), the cleaved CASPASE-1/pro-CASPASE-1 ratio, the cleaved GSDMD/GSDMD ratio, and the pro-IL-1β/IL-1β ratio measured in left ventricular tissues obtained from sham-operated and TAC-operated *Acsl4* F/F and *Acsl4* KO mice (*n* = 5 mice/group). Western blot analysis (**g**) and quantification (**h**) of NLRP3, ASC, the cleaved CASPASE-1/pro-CASPASE-1 ratio, the cleaved GSDMD/GSDMD ratio, and the pro-IL-1β/IL-1β ratio measured in left ventricular tissues obtained from vehicle-treated TAC-operated wild-type (WT) mice, 12-HETE‒treated TAC-operated WT mice and 12-HETE + Fer-1‒treated TAC-operated WT mice (*n* = 5 mice/group). **i** Left ventricular tissues were obtained from vehicle-treated and 12-HETE‒treated TAC-operated WT and *Nlrp3* KO mice and stained with WGA (green); Evans blue‒positive cardiomyocytes are indicated in red, and the nuclei were counterstained with DAPI (blue). Scale bar, 50 μm. **j** Summary of Evans blue–positive cells in the indicated mice (*n* = 5 mice/group). **k** Summary of the heart weight/tibia length ratio measured in vehicle-treated and 12-HETE‒treated TAC-operated WT and *Nlrp3* KO mice (*n* = 5–6 mice/group). *P < 0.05, **P < 0.01, ***P < 0.001, and ns, not significant (one-way or two-way ANOVA followed by Tukey’s multiple comparisons test)

To examine the molecular mechanisms that underlie the progression of HF via ACSL4-mediated ferroptosis, we performed RNA-seq analysis in sham-operated and TAC-operated *Acsl4* F/F and *Acsl4* KO mice (Fig. 6a). Gene enrichment analysis between these two groups revealed enrichment of genes in both IL-1 signaling and IL-1β production (Fig. 6b). Moreover, we found that the expression of *Il1b* and *Nlrp3* (which encode the cytokine IL-1β and the pyrin-like protein NLR family pyrin domain containing 3, respectively) were significantly upregulated in TAC-induced cardiac hypertrophy in *Acsl4* F/F mice, but not in *Acsl4* KO mice (Fig. 6c).

IL-1β is produced by the cleavage of its precursor pro-IL-1β, a process activated by the canonical inflammasome consisting of a cytosolic sensor such as NLRP3, an adaptor protein such as ASC (apoptosis-associated speck-like protein containing a CARD), and an effector caspase such as pro-caspase-1 (Fig. 6d)^38^. Given our finding that both *Il1b* and *Nlrp3* are significantly downregulated in TAC-operated *Acsl4* KO mice compared to TAC-operated *Acsl4* F/F mice, we measured the both pro-IL-1β and IL-1β in sham-operated and TAC-operated mice at protein levels. Interestingly, we found that both IL-1β protein levels and the IL-1β/pro-IL-1β ratio were increased in TAC-operated *Acsl4* F/F mice compared to sham-operated *Acsl4* F/F mice, but were significantly lower in TAC-operated *Acsl4* KO mice compared to TAC-operated *Acsl4* F/F mice (Fig. 6e, f). Moreover, we found that TAC-operated *Acsl4* F/F mice had increased pyroptotic signaling, including increased Nlrp3 levels, an increase in the cleaved CASPASE-1/pro-CASPASE-1 ratio, and an increase in the ratio between cleaved GSDMD and GSDMD (Gasdermin D), and again these increases were significantly reduced in TAC-operated *Acsl4* KO mice (Fig. 6e, f). We then tested whether blocking IL-1β signaling using an IL-1β‒neutralizing antibody can protect against the pathological effects of TAC-induced pressure overload. We therefore gave control and *Acsl4* TG mice weekly i.p. injections of anti‒IL-1β antibody and then examined heart structure and cardiac function 3 weeks after TAC surgery (Supplementary Fig. 11a). We found that neutralizing IL-1β in *Acsl4* TG mice significantly reduced TAC-induced cardiac hypertrophy (Supplementary Fig. 11b), improved cardiac systolic performance (Supplementary Fig. 11c, d), prevented cardiomyocyte hypertrophy (Supplementary Fig. 11e), and reduced all three markers of cardiac hypertrophy (Supplementary Fig. 11f, g). These findings support the notion that increased production of the cytokine IL-1β plays an important role in pressure overload-induced, ACSL4-dependent ferroptosis in cardiomyocytes.

Given the finding that knocking out *Acsl4* in cardiomyocytes reduced TAC-induced 12-HETE production and lipid peroxidation, we then asked whether lipid peroxidation plays a role in the 12-HETE-mediated activation of pyroptotic signaling. We found that compared to vehicle-treated TAC-operated WT mice, 12-HETE‒treated TAC-operated WT mice had increased pyroptotic signaling (Fig. 6g, h), increased heart mass (Supplementary Fig. 12a), and increased ferroptosis markers such as higher MDA levels, a lower GSH/GSSG ratio, and higher *Ptgs2* mRNA levels (Supplementary Fig. 12b–d); moreover, all of these 12-HETE–induced effects were reduced by Fer-1 treatment, indicating that pressure overload-induced ferroptosis activates the pyroptotic pathway.

Lastly, to confirm the effects of 12-HETE in vivo, we generated global *Nlrp3* knockout (*Nlrp3* KO) mice. At baseline, we found no clear difference in cardiac function or structure between WT mice and *Nlrp3* KO mice (Supplementary Fig. 13). We then performed TAC surgery on *Nlrp3* KO and WT mice and measured heart mass and cardiac Evans blue uptake in order to examine cardiac hypertrophy and cardiac cell death, respectively. We found that compared to 12-HETE‒treated TAC-operated WT mice, 12-HETE‒treated TAC-operated *Nlrp3* KO mice had reduced cardiac cell death (Fig. 6i, j) and reduced cardiac hypertrophy (Fig. 6k).

## Discussion

Here, we report that both the production of PEs and ACSL4 expression significantly increase in pressure overload-induced heart failure. We also show that overexpressing Acsl4 selectively in mouse cardiomyocytes exacerbates TAC-induced HF via ferroptosis, whereas both pharmacologically blocking and genetically knocking out Acsl4 reduces TAC-induced HF. With respect to the underlying mechanism, we show that ACSL4-dependent ferroptosis drives the activation of pyroptosis in response to cardiac hemodynamic stress.

Among the five known ACSL family members, we identified ACSL4 as the major player in pressure overload-induced HF. Cardiac tissue consumes considerably more energy than most other organs, and fatty acids are used to produce ~60–90% of the ATP synthesized in the heart.^39^ via a process that requires the enzymatic activity of ACSL family members. Tsushima *et al*. previously reported that mice overexpressing Acsl1 in cardiomyocytes develop cardiac hypertrophy by 12 weeks of age.^16^ In contrast, Goldenberg et al. subsequently reported that mice in which *Acsl1* expression is driven by the cardiomyocyte-specific *Myh6* promoter had reduced TAC-induced cardiac hypertrophy and dysfunction compared to controls.^17^ Interestingly, using the mouse model of TAC-induced pressure overload, we found that Acsl4 is significantly upregulated—while Acsl1 is downregulated—following TAC, suggesting that these two family members have distinct functions and/or substrate preferences. Arachidonic acid (C20:4) is the preferred substrate for ACSL4,^40^ and we found that this fatty acid was one of the most strongly increased PEs in response to hypertrophic stimuli. Interestingly, we also found that overexpressing Acsl4 selectively in cardiomyocytes promotes cardiac dysfunction by directly increasing lipid peroxidation. Given that arachidonic acid contains bis-allylic hydrogen atoms, which are susceptible to lipid peroxidation, these results indicate that ACSL4 plays a more prominent role in regulating lipotoxicity than ACSL1, at least in our TAC-induced mouse model.

We previously reported that mouse models of doxorubicin- and ischemia/reperfusion-induced cardiomyopathy develop cardiac ferroptosis, an iron-dependent form of programmed cell death.^30^ In our current study, we found that blocking ferroptosis reduces pressure overload-induced HF. The ferroptosis pathway lies at the intersection of metabolic processes involving iron, amino acids, and lipids,^21,41^ and we previously reported that mice lacking ferritin H (the heavy chain of the iron-binding protein ferritin) in cardiomyocytes have decreased cardiac iron levels and increased oxidative stress, which accelerates iron-induced cardiac injury by triggering ferroptosis.^30^ In that same study, we also found that overexpressing *Slc7a11*, which encodes the cystine/glutamate antiporter xCT, significantly reduced the effects of iron-induced cardiac injury.^30^ On the other hand, Zhang et al. recently reported that *Slc7a11* knockout mice have increased angiotensin II-induced cardiac hypertrophy and cardiac dysfunction.^42^ Moreover, mice lacking Ncoa4 (nuclear receptor coactivator 4, which mediates ferritinophagy) were reported to have reduced TAC-induced HF.^43^ Here, we provide the first evidence that ACSL4-mediated lipid metabolism drives the progression of HF via ferroptosis. Given these findings, we suggest that ferroptosis may serve as a common pathogenic mechanism driving the development of HF.

An interesting finding from our study is that pyroptotic signaling appears to play a key role in ACSL4-mediated ferroptosis during HF. Pyroptosis results in activation of the NLRP3 inflammasome, which leads to the caspase-1-dependent release of IL-1β and GSDMD-mediated pyroptotic cell death.^44^ Here, we show that activation of NLRP3 is involved in ACSL4-induced cardiac dysfunction during TAC-induced pressure overload, with activation of caspase-1, cleavage of IL-1β and GSDMD, and induction of cardiomyocyte death. We also found that neutralizing IL-1β slows the progression of HF induced by overexpressing Acsl4. Moreover, previous studies found that healthy mice injected with IL-1β develop transient systolic dysfunction,^45^ and several clinical trials targeting IL-1 (e.g., the CANTO,^46^ REDHART,^47^ and ADHF^48^ trials) found protective effects against cardiac dysfunction and/or improved exercise tolerance in patients with HF. Consistent with these findings, Suetomi et al. found that both *Il1b* and *Nlrp3* are upregulated in pressure-overloaded mice,^49^ and other studies found that activation of the NLRP3 inflammasome complex results in IL-1β production^50^ and pyroptotic cell death.^51,52^ Here, we show that loss of Nlrp3 reduces TAC-induced cardiac cell death, but we did not explicitly demonstrate that this effect is mediated by GSDMD-mediated pyroptosis; therefore, although we claim that pyroptotic signaling is involved in TAC-mediated HF, further works are needed to examine whether pyroptosis also plays a role. Nevertheless, we show that ferroptosis activates pyroptotic signaling in cardiomyocytes during the development of HF. Previously, Kuwata *et al*. showed that stimulating bone marrow-derived macrophages with lipopolysaccharide increased the release of prostaglandins, and this effect was significantly enhanced in *Acsl4* knockout cells.^53^ Given that canonical pyroptosis is an innate immune effector, further works are needed to determine whether TAC-induced ferroptosis in cardiomyocytes also engages pyroptotic signaling in cardiac immune cells.

Notably, we also found that ALOX-mediated 12-HETE production plays a functional role in ACSL4-induced cardiac hypertrophy. ALOX proteins are belonging to non-heme iron-containing enzymes that mediate lipid peroxidation,^54^ and the oxidation of arachidonic acid by ALOX generates bioactive oxylipins, including leukotrienes, lipoxins, and hydroxyeicosatetraenoic acids.^37,55^ Our finding that ACSL4-dependent ferroptosis activates ALOX12 is consistent with recent reports.^56^ Interestingly, another recent study found that both ALOX15 and its intermediate metabolite 15-HETE aggravate myocardial ischemia/reperfusion injury by activating ferroptosis in cardiomyocytes,^57^ and both ALOX12 and ALOX15 have been reported to generate 12-HETE.^58^ Moreover, we found that 12-HETE levels are increased during hemodynamic stress and are reduced in the absence of Acsl4. Importantly, 12-HETE activates the pyroptotic signaling pathway, suggesting that this metabolite may serve as the bridge linking ferroptosis to pyroptosis. Triacylglycerol levels change in TAC-induced heart failure, and this may be caused by lipolysis, a catabolic process in which triacylglycerol is broken down into fatty acids and glycerol.^59^ The resulting fatty acids serve as an energy substrate to be utilized for fatty acid oxidation and subsequent ATP production. Moreover, the ACSL family participates in the production to form fatty acyl-CoA from fatty acids, a key step to fatty acid oxidation.^29^ This may also explain our finding that hypertrophic stimuli increase Acsl4 expression and increase mitochondrial oxygen consumption rates. Given the specific phenotype observed in mice with TAC-induced HF, these data may not necessarily reflect all types of HF in patients; nevertheless, aortic banding‒induced pressure overload (i.e., TAC) in mice is a commonly used model for studying HF caused by the burden due to increased cardiac afterload. In addition, systemically inhibiting ACSL4 affects virtually all tissues and cell types—not just cardiomyocytes—and may therefore have other effects that are comparable to or greater than cardiac-specific *Acsl4* knockout.

Despite recent advances in pharmacological therapies and medical devices, the prognosis for patients with HF remains poor. Therefore, an important goal in improving patient outcome is to better understand the pathophysiological processes that underlie HF. Here, we report that HF triggered by hemodynamic stress is accompanied by lipid metabolism‒induced ferroptosis. We also identified ACSL4 as the major player upstream of ferroptosis, showing that it drives cardiac hypertrophy by activating the pyroptotic pathway. These results may have clinical relevance, as we found that both pharmacologically blocking and genetically silencing Acsl4 significantly improved cardiac function in our mouse model of pressure overload-induced HF, suggesting that ACSL4 may represent a possible target for future therapeutic interventions designed to treat and/or prevent heart failure.

## Materials And methods

The data, methods, and study materials are available from the corresponding authors upon reasonable request. Expanded Methods are provided in the Supplemental Materials.

### Animals

All animal research were approved by the Zhejiang University Animal Care and Use Committee (approval number #29207). The mice were housed under a 12-h dark/light cycle (with the lights on from 6 am to 6 pm) with unlimited access to water and chow. Mice carrying the *Acsl4*^*flox*^ allele were generated at Shanghai Model Organisms using homologous recombination. In brief, a donor vector was constructed using In-Fusion cloning, containing a 3.8-kb 5′ homology arm, a 0.8-kb floxed region, and a 3.5-kb 3′ homology arm. After injecting the *Cas9* mRNA, gRNA, and donor vector, zygotes containing correctly targeted clones were identified. LoxP sites were inserted into introns flanking exon 5 in the mouse *Acsl4* gene. *Acsl4*^*flox/flox*^ mice were crossed with Myh6-Cre mice to generate cardiomyocyte-specific *Acsl4* knockout (*Acsl4* KO) mice; using this approach expression of the Cre recombinase transgene was driven by the *Myh6* promoter, which is specifically expressed in cardiomyocytes; thus, Cre recombinase targets and excises the DNA sequence between two LoxP site selectively in cardiomyocytes. To generate *Acsl4*-expressing transgenic (*Acsl4* TG) mice, the CAG-flox-STOP-flox-Acsl4-IRES-tdTomato-Wpre-pA vector was inserted into mouse chromosome 11: 3,245,259 using CRISPR-mediated homologous recombination (Shanghai Model Organisms). By crossing transgene-positive mice with Myh6-Cre mice, we overexpress Acsl4 selectively in cardiomyocytes. Global *Nlrp3* knockout mice were kindly provided by Prof. Di Wang (Zhejiang University) and were generated as previously reported.^60^ Myh6-Cre mice were obtained from The Jackson Laboratory (strain #011038) and have been described previously.^30^ The PCR primers used for genotyping are listed in Table S1. Unless indicated otherwise, male mice 10–12 weeks of age were used.

#### Transverse aortic constriction

TAC surgery was performed as previously described.^61^ In brief, male mice were anesthetized with a cocktail of ketamine (100 mg/kg body weight) and xylazine (5 mg/kg body weight) and then placed on a warming pad to maintain body temperature at 34–37 °C. The left chest was opened at the second intercostal space, and the thoracic aorta was identified and ligated using a 28-gauge needle. The entire surgical procedure was applied to sham-operated mice, but the aorta was not ligated. We randomly chosed the mice before the surgery, and the surgeon was blinded with respect to the genotype and/or treatment group.

#### Drug administration

##### Ferrostatin-1 (Fer-1)

One day before sham or TAC surgery, mice received an i.p. injection of 1 mg/kg body weight ferrostatin-1 (Fer-1; Selleck, S7243) or vehicle and for 21 days after surgery as previously reported.^43^

##### Anti-IL-1β antibody

As previously described,^62,63^ the anti-IL-1β antibody or a control IgG (10 mg/kg body weight/week) was injected i.p. in *Acsl4* TG and Ctrl mice. The mice were then subjected to TAC surgery, and weekly injections of antibody were continued for an additional 21 days.

##### PRGL493

The mice were treated with either PRGL493 (MedChemExpress, HY-139180) or vehicle as previously reported.^64^ In brief, one day before sham or TAC surgery and for 21 days after surgery, the mice received i.p. injections of PRGL493 (250 μg/kg body weight/day) or vehicle.

##### Pioglitazone

As previously reported,^65^ pioglitazone (Selleck, S2590) treatment was initiated 1 week before TAC surgery and continued for 3 weeks by addition to the food at a concentration of 0.01% (wt/wt).

##### 12-HETE

12-HETE was purchased from Sigma (H7768). One day before TAC surgery and for 21 days after surgery, the mice received daily intranasal injections of 12-HETE (200 ng in 6 μl PBS) or vehicle, as described previously.^66^

#### Echocardiography

Cardiac systolic function was evaluated in conscious, gently restrained mice using a Vevo 1100 system (Visual Sonics, MS400 probe) or Vevo 3100 system (Visual Sonics, MX400 probe). At the level of the left ventricular papillary muscles, M-mode recordings were captured. The images were analyzed in order to calculate the heart rate, left ventricular end-diastolic internal dimension (LVIDd), left ventricular end-diastolic internal dimension (LVIDs), and left ventricular end-diastolic posterior wall thickness (LVPWd). Fractional shortening, expressed as a percentage (FS%), was calculated using the formula [(LVIDd-LVIDs)/LVIDd].

#### Histology

The mice were sacrificed, and the whole heart was harvested, fixed in 4% paraformaldehyde for 48 h, embedded in paraffin, and sectioned at 5-μm thickness. The sections were stained with Masson’s trichrome stain at the core facility platform at Zhejiang University. Wheat germ agglutinin (WGA, Invitrogen, W21405) staining was also performed and used to analyze cross-sectional area of the cardiomyocytes. Cardiomyocyte size was quantified by choosing cells with a circularity coefficient >0.7; the circularity coefficient was calculated using the formula (4π*A/P^2^), where *A* is the area and *P* is the perimeter, as described previously.^61^

#### Evans blue studies

Evans blue dye was purchased from Sigma (E2129) and dissolved in PBS to a 1% (w/v) solution. The mice received an i.p. injection of 1% Evans blue solution (1 ml/g body weight) as previously reported.^67^ After 24 h, the mice were sacrificed and the heart was removed, frozen in OCT compound (SAKURA, 4583), and sectioned at 10-μm thickness using a CryoStar NX50 microtome (Thermo Fisher Scientific). For analysis, the sections were warmed to room temperature, fixed in pre-chilled 100% methanol for 10 min, stained with WGA (Invitrogen, W21405) for 30 min, and then fixed with ProLong Gold Antifade with DAPI (Thermo Fisher Scientific, P36931). A coverslip was applied and fixed to the slide using nail polish, and the sections were imaged using an Olympus fluorescence microscope.

### Statistical analysis

Unless indicated otherwise, all summary data are presented as the mean ± SEM and were analyzed using Prism 9 (GraphPad Prism). Details statistical analyses are provided in the figure legends and in the Supplemental Materials.

## Supplementary information

SUPPLEMENTAL MATERIALBi et al Supplementary Materials

ORIGINAL GEL

## Data Availability

The data that support the findings of this study are available from the corresponding author upon request.
