# Supplementary material for: Characterization of ferroptosis-triggered pyroptotic signaling in heart failure
Source: Signal Transduct Target Ther. 2024 Sep 25;9:257. doi: 10.1038/s41392-024-01962-6 (PMC11427671; doi:10.1038/s41392-024-01962-6)
Supplement: Supplementary file 1 — SUPPLEMENTAL MATERIALBi et al Supplementary Materials [file 41392_2024_1962_MOESM1_ESM.pdf]

SUPPLEMENTAL MATERIALS for

Characterization of ferroptosis-triggered pyroptotic signaling in heart failure

Xukun Bi[†], Xiaotian Wu[†], Jiaqi Chen, Xiaoting Li, Yangjun Lin, Yingying Yu, Xuexian Fang, Xihao Cheng, Zhaoxian Cai, Tingting Jin, Shuxian Han, Meihui Wang, Peidong Han, Junxia Min*, Guosheng Fu*, Fudi Wang*

*Correspondence to:

Fudi Wang (fwang@zju.edu.cn) or Guosheng Fu (fugs@zju.edu.cn) or Junxia Min (junxiamin@zju.edu.cn)

This PDF file includes:

Detailed Methods

Supplementary Fig. 1 to Supplementary Fig. 14

Supplementary Table 1

DETAILED METHODS

Transmission electron microscopy

Phenylephrine-treated neonatal rat ventricular myocytes (NRVMs) were rapidly digested in trypsin, and then immediately fixed in 2.5% (w/v) pentanediol in PBS overnight. Cardiac left ventricular samples were also fixed in 2.5% pentanediol overnight. The Center for Cryo-Electron Microscopy at Zhejiang University post-fixed, embedded, cut, and mounted the samples, and thin sections (85-nm thickness) were viewed using a Talos L120C transmission electron microscope (Thermo Fisher Scientific).

RNA isolation and real-time qPCR

Cardiac RNA was extracted using TRIzol reagent, and 150-250 ng RNA was reverse-transcribed using the PrimeScript RT reagent Kit (Takara). The resulting cDNA was then diluted 10-fold with ddH_2O, and real-time qPCR was performed using SYBR Green Supermix (Bio-Rad) in a LightCycler 480 (Roche). The fold difference in relative mRNA expression was calculated using the $2^{-\Delta\Delta Ct}$ method, with *18S* rRNA serving as an internal control. The PCR primers used for RT-qPCR are listed in Table S1.

RNA-seq analysis

A total of 2 μg RNA per left ventricular sample was used as input material. These experiments were performed at Annoroad Gene Technology using three independent biological replicates. Sequencing libraries were generated using the NEBNext Ultra RNA Library Prep Kit for Illumina (E7530L, New England Biolabs, Ipswich, MA) in accordance with the manufacturer's instructions. RNA concentration of the library was measured using a Qubit RNA Assay Kit with Qubit 3.0 (Thermo Fisher Scientific), and then diluted to 1 ng/μl. Clustering of the index-coded samples was performed on a cBot cluster generation system using the HiSeq PE Cluster Kit v4-cBot-HS (Illumina). The libraries were then

sequenced on an Illumina platform, and 150-bp paired-end reads were generated. Raw reads were filtered to ensure data quality for further analysis. The *Mus musculus* reference genome and the annotation file were downloaded from ENSEMBL database. For pathway enrichment analysis, a *P*-value ≤0.05 and a fold change of >2 or <0.5 were used as the threshold to determine significant enrichment of the gene sets. The dataset was uploaded to ArrayExpress (accession numbers #E-MTAB-14383).

Targeted lipidomics

Metabolites were extracted from 20 mg of each left ventricular sample. After adding 400 μl water, each sample was vortexed for 60 s, homogenized at 45 Hz for 4 min, and then sonicated for 5 min in an ice-water bath; this homogenization/sonication cycle was repeated 3 times. Next, 50 μl of homogenate was mixed with 150 μl water and 480 μl extract solution containing an internal standard. After vortexing for 60 s, the samples were sonicated for 10 min in an ice-water bath. The samples were then centrifuged at 4°C for 15 min at 3000 rpm, and 250 μl of supernatant was transferred to a fresh tube; the remaining volume of each sample was combined with 250 μl MTBE (methyl-tertiary-butyl ether), vortexed, sonicated, and centrifuged, and another 250 μl of supernatant was removed. After repeating this extraction, 750 μl of the collected supernatants was combined and dried in a vacuum concentrator at 37°C. The dried samples were reconstituted in 100 μl resuspension buffer consisting of dichloromethane:MeOH:H_2O at a 60:30:4.5 ratio; the samples were then vortexed for 30 s and sonicated for 10 min in an ice-water bath. The samples were then centrifuged at 4°C for 15 min at 12,000 rpm, and 40 μl of supernatant was transferred to a fresh glass vial for LC/MS analysis. The dataset was uploaded to Mendeley Data (DOI: 10.17632/bfry768hk7.1).

UPLC separation was carried out using an ExionLC series UHPLC system (Sciex). The mobile phase A consisted of 40% water, 60% acetonitrile, and 10

mmol/L ammonium formate. The mobile phase B consisted of 90% isopropanol, 10% acetonitrile, and 10 mmol/L ammonium formate. The column temperature was 40°C, the auto-sampler temperature was 6°C, and the injection volume was 2 μl. A QTrap 6500+ mass spectrometer (Sciex) was used for assay development, with the following ion source parameters: ion spray voltage: +5500/-4500 V; curtain gas: 40 psi, temperature: 350°C, ion source gas 1: 50 psi, ion source gas 2: 50 psi; and DP: ±80V.

Biobud v2.07 software was used to quantify the target compounds. The absolute content of individual lipids was calculated based on an internal standard using the peak area and actual concentration of an identical lipid internal standard.

Analysis of oxylipins

For each sample, 20 mg of left ventricular tissue was homogenized in 200 μl oxidized lipid extract and then spiked with 20 μl of 1 μM internal standard mixture. All eicosanoids and deuterated internal standards were purchased from Cayman Chemical. The stock solutions of standards were prepared at 5 μg/ml in MeOH. The samples were vortexed for 10 min, centrifuged at 4°C for 10 min at 5000 rpm, and supernatant was transferred to a fresh tube; this extraction step was repeated once, and all supernatants were collected. The eicosanoids in the supernatants were extracted using Poly-Sery MAX SPE columns (Anpel Laboratory Technologies); the eluent was dried under vacuum and then re-dissolved in 100 μl of methanol/water (1:1, v/v) for LC/MS analysis.

UPLC separation was performed using an ExionLC system as follows. The mobile phase A consisted of water with 0.04% acetic acid. The mobile phase B consisted of acetonitrile with 0.04% acetic acid. The column temperature was 40°C, and the injection volume was 10 μl. A QTrap 6500+ mass spectrometer was used for assay development with the following parameters: ion spray

voltage: -4500 V; curtain gas: 35 psi; and temperature: 550°C.

Analyst v1.6.3 software (Sciex) and MultiQuant v3.0.3 software (Sciex) were used to quantify the metabolites.

Isolation and culture of rat and mouse cardiomyocytes

Rat NRVMs were prepared from the hearts of newborn (1-3-day-old) Sprague-Dawley rats (Shanghai Jihui Laboratory Animal Care Company). In brief, the ventricles were minced in vials and digested with 1 mg/ml collagenase II (Gibco, 2167645) and 1 mg/ml trypsin (Solarbio, Beijing, China) at 37°C. After pre-plating for 90 min to remove non-cardiomyocytes, the cardiomyocytes were plated at a density of 1250 cells/mm^2 in plating medium consisting of DMEM/M199 (3:1; Cienry, Huzhou, China), 10% (v/v) fetal bovine serum (FBS, GeminiBio, Woodland, CA), and 0.1 mmol/L bromodeoxy uridine (BrdU). After 24 hours, the cells were washed and cultured in low-serum medium consisting of DMEM/M199 (3:1), 1% (v/v) FBS, and 0.1 mmol/L BrdU. Twenty-four hours later, the cells were washed again and cultured in serum-free medium consisting of DMEM/M199 (3:1) and 0.1 mmol/L BrdU.

Adult mouse cardiomyocytes were isolated from the hearts of 8-12-week-old male mice. In brief, the mice were sacrificed, the hearts were immediately harvested, cannulated, and then digested by perfusion with modified Krebs-Ringer buffer consisting of 10 mM HEPES, 1.19 mM KH_2PO_4, 35 mM NaCl, 4.75 mM KCl, 16 mM Na_2HPO_4, 10 mM glucose, 134 mM sucrose, and 25 mM $NaCO_3$ (pH 7.4) containing 0.8 mg/ml collagenase II (Worthington). After digestion for 15 min at 37°C, the left ventricular was isolated and cut into small pieces. After filtering through a cell strainer, the cardiomyocytes were enriched by sedimentation.

Immunofluorescence

NRVMs were seeded on round coverslips in 24-well plates. After the indicated treatment, the cells were washed 3 times with pre-cooled PBS, and then fixed with 4% paraformaldehyde for 10 min. After three washes with PBS for 5 min each, the cells were permeabilized with TBS-T (Tris-buffered saline containing 0.25% Triton X-100) for 10 min at room temperature. The TBS-T was removed, and the cells were washed 3 times with PBS. The cells were then blocked with PBS containing 1.5% (v/v) normal goat serum and 1% (w/v) BSA for 30 min, followed by incubation with α-actinin primary antibody (Sigma, A7732; 1:100). After washing 3 times with PBS, the cells were incubated with goat anti-mouse Alexa 568 (Thermo Fisher Scientific, A-11031) for 1 hour. The NRVMs were then fixed with ProLong Gold Antifade with DAPI (Thermo Fisher Scientific, P36931). The coverslip was then mounted to a microscope slide using nail polish, and the cells were imaged using an Olympus fluorescence microscope.

Adult mouse cardiomyocytes were seeded on confocal dishes. The cells were washed 3 times with pre-cooled PBS, and then fixed with 4% paraformaldehyde for 10 min. After three washes with PBS for 5 min each, the cells were permeabilized with TBS-T (Tris-buffered saline containing 0.25% Triton X-100) for 10 min at room temperature. The TBS-T was removed, and the cells were washed 3 times with PBS. The cells were then blocked with PBS containing 1.5% (v/v) normal goat serum and 1% (w/v) BSA for 30 min, followed by incubation with Acsl4 primary antibody (Santa Cruz, sc-365230). After washing 3 times with PBS, the cells were incubated with goat anti-mouse Alexa 568 (Thermo Fisher Scientific, A-11031) for 1 hour. The cells were then incubated with DAPI. After washing 3 times with PBS, and the cells were imaged using a Nikon A1R confocal microscope.

Western blot analysis

Where indicated, left ventricular tissues (20-50 mg) or NRVMs were lysed in RIPA buffer supplemented with protease inhibitors and phosphor-STOP

(Roche). The lysates were then centrifuged at 14,000 rpm for 10 min at 4°C. Equal amounts of protein (10-20 µg) were then separated by SDS-PAGE (Criterion, Bio-Rad), and then transferred to nitrocellulose membranes for immunoblotting using the following primary antibodies: Acsl4 (Santa Cruz, sc-365230), Acsl5 (Santa Cruz, sc-365478), Acsl1 (Cell Signaling Technology, #4047S), Gpx4 (Abcam, ab125066), Rcan1 (Sigma, D6694), Anf (Abcam, ab180649), β-MHC (Proteintech, 22280-1-AP), Alox12 (Santa Cruz, sc-365194), NLRP3 (AdipoGen Life Sciences, AG-20B-0044), Caspase-1 (AdipoGen Life Sciences, AG-20B-0042), ASC (AdipoGen Life Sciences, AG-25B-0006), GSDMD (Abcam, ab209845), IL-1β (R&D Systems, AF-401-NA), and GAPDH (Fitzgerald, 10R-G109A), followed by IRDye 800CW goat anti-rabbit (Li-Cor Biosciences, 926-32211) and IRDye 680RD goat anti-mouse (Li-Cor Biosciences, 926-68070) secondary antibodies. Fluorescence was detected using an Odyssey scanner (Li-Cor Biosciences).

Enzyme-linked immunosorbent assays

Where indicated, left ventricular tissues (20-50 mg) or NRVMs were lysed and centrifuged at 250*g* for 10 min at 4°C to remove cell debris, and the concentration of IL-1β protein in the lysates was measured immediately using an IL-1β Mouse Uncoated ELISA Kit (Invitrogen, 88-7013-88) in accordance with the manufacturer's recommendations. Protein content was used to normalize all treatments. Mouse serum Troponin I protein levels were measured using a Mouse Cardiac Troponin I ELISA Kit (Invitrogen, EEL112).

siRNA-mediated knockdown

NRVMs were seeded in 6-well plates containing 2 ml of serum-free plating medium consisting of DMEM/M199 (3:1); 24 hours later, the cells were transfected with either a scrambled siRNA (*siCtrl*) or an siRNA (Sigma) directed against *Acsl4* (*siAcsl4*) using Lipofectamine RNAiMAX (Thermo Fisher Scientific, 13778075). In brief, 160 pmol of siRNA was diluted in 250 µl Opti-

MEM, and 8 μl RNAiMAX was diluted in 250 μl Opti-MEM; the two solutions were then combined to form the siRNA-lipid complex. After incubation for 5-10 min at room temperature, the siRNA-lipid complex was added to the cells; 6 hours after transfection, the cells were washed and then cultured in serum-free plating medium. The cells were used for experiments after an additional 48 hours of incubation.

Oxygen consumption

Oxygen consumption was measured in NRVMs using the XFe96 Mito Stress Test (Agilent, 103015-100) in accordance with the manufacturer's instructions. In brief, 10,000 NRVMs were seeded in XFe96 cell culture plates. The cells were then treated with siRNA and either phenylephrine or vehicle, after which 1.5 μM oligomycin, 2 μM FCCP (carbonyl cyanide p-trifluoromethoxyphenylhydrazone), and 0.5 μM antimycin A/rotenone were added to the reagent ports. Oxygen consumption was measured automatically using Seahorse Wave (Agilent).

Adenovirus infection

To overexpress Acsl4, an adenovirus expressing *Acsl4* (Ad-Acsl4) was used to infect NRVMs (MOI: 5-15); adenovirus expressing *GFP* (Ad-GFP) was used as a negative control. The culture medium was replaced 6 hours after infection, and the cells were treated with 50 μM phenylephrine and/or 5 mM *N*-acetyl-L-cysteine (Sigma, A9165) for 24 hours.

MDA, GSH, and GSSG measurements

Cardiac malondialdehyde (MDA) levels were measured using the Lipid Peroxidation MDA Assay Kit (Beyotime, S0131), and cardiac GSH and GSSG were measured using the GSH and GSSG Assay Kit (Beyotime, S0053) in accordance with the manufacturer's instructions.

Flow cytometry

NRVMs were seeded in 6-well plates and transfected with siRNA as described above. Following phenylephrine treatment, a stock solution of 1.5 μM BODIPY-C11 was added to the cells, and the cells were cultured for 20 min. The cells were washed twice with 1 ml PBS, digested with 0.05% trypsin without EDTA, and the trypsin was quenched by the addition of FBS. The cells were concentrated, resuspended in 400 μl PBS, and analyzed using an ACEA Novocyte flow cytometer (Agilent).

To detect dead cells, cells treated with siRNA and/or phenylephrine (including floating dead cells) were stained with 1 μg/ml propidium iodide (PI; Thermo Fisher Scientific, P1304MP), and the percentage of PI–positive (i.e., dead) cells was analyzed using flow cytometry.

To measure intracellular ROS, treated cells were incubated with 5 mM 2',7'-dichlorofluorescein diacetate (H_2DCFDA, Sigma, D6883) for 30 min. The cells were then harvested, resuspended in PBS, and analyzed using flow cytometry.

To measure mitochondrial membrane potential, treated NRVMs were incubated for 30 min with 1 μg/ml JC-1 (Beyotime, C2006). The cells were then harvested and resuspended in PBS. In healthy cells, JC-1 accumulates in the mitochondria to form red fluorescent aggregates; mitochondrial depolarization leads to the formation of green fluorescent monomers. We then performed flow cytometry and used the reduction in red fluorescence to measure mitochondrial membrane depolarization.

Statistics

Student's *t*-test (2-tailed) was used to compare two groups. Multiple groups were compared using a one-way or two-way ANOVA followed by Tukey's multiple comparisons test. Differences were considered significant at $P<0.05$.

SUPPLEMENTARY DATA

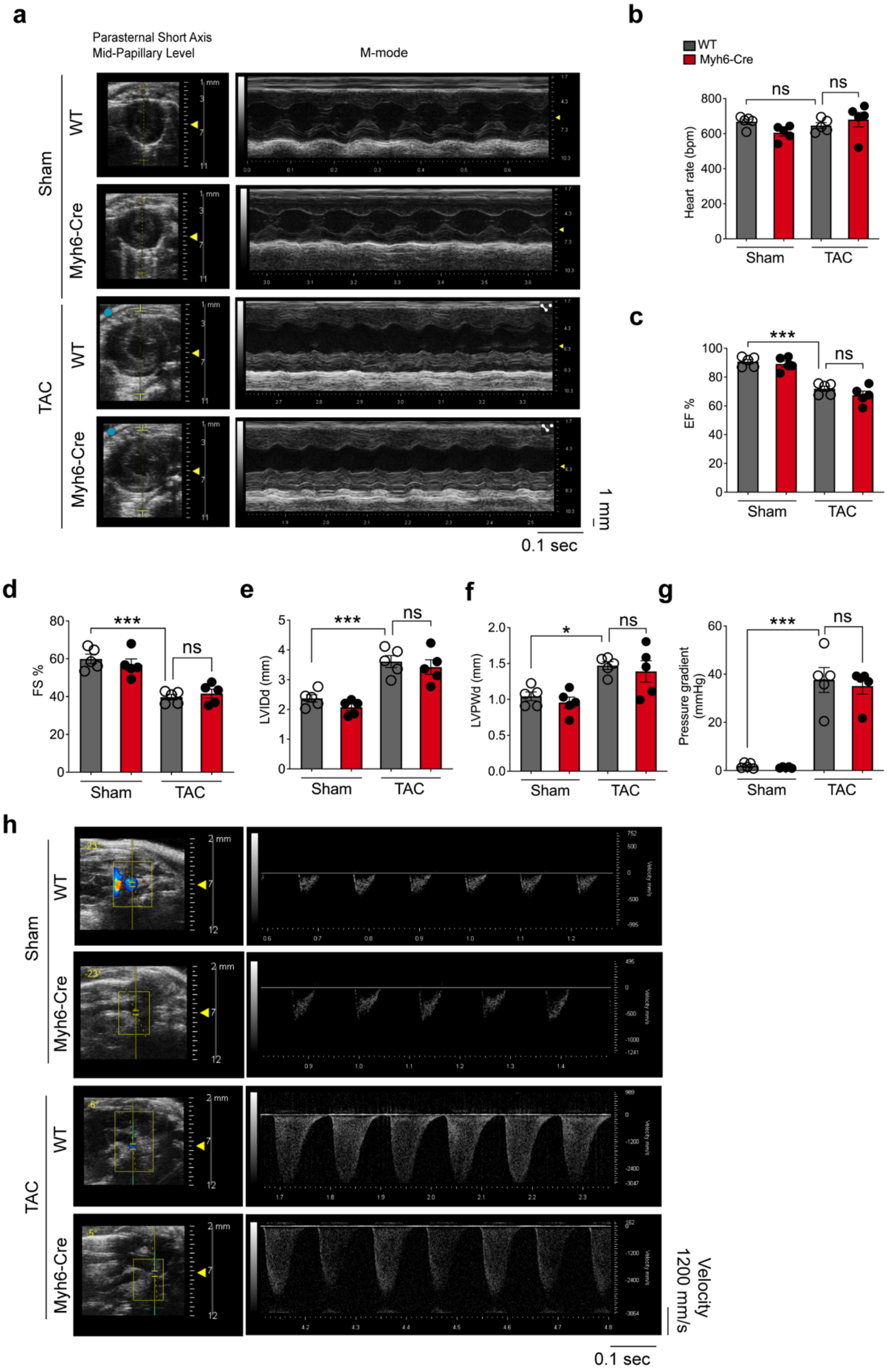

Supplementary Fig. 1 TAC induces heart failure in mice. a, Representative echocardiography images measured in sham-operated and TAC-operated wild-type (WT) and Myh6-Cre mice. **b-f**, Summary of heart rate (b), ejection fraction (c), fractional shortening (d), left ventricular end-diastolic internal dimension (e), and left ventricular posterior wall thickness at diastole (f) in the indicated groups (n=5 mice/group). **g** and **h**, Doppler imaging was performed in order to measure peak aortic velocity across the TAC site. The pressure gradient was when calculated using $4 \times V_{max}^2$, according to the modified Bernoulli equation (n=5 mice/group). In this and subsequent figures, unless indicated otherwise summary data are presented as the mean ± SEM. **P*<0.05, ****P*<0.001, and ns, not significant (two-way ANOVA).

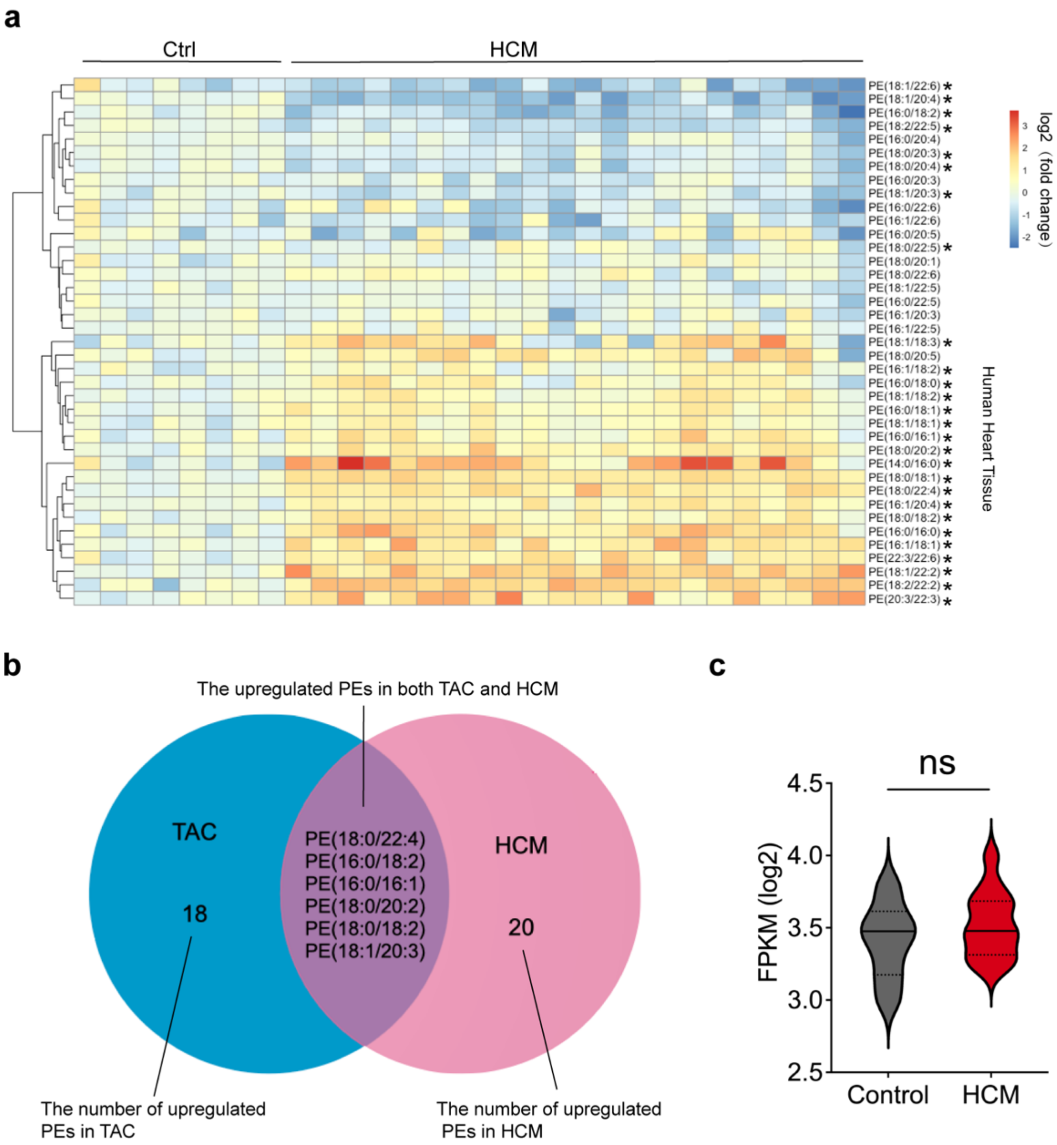

Supplementary Fig. 2 Overview of phosphatidylethanolamine metabolites in heart tissues obtained from patients with hypertrophic cardiomyopathy. **a**, Heatmap depicting the relative abundance of the indicated phosphatidylethanolamine (PE) metabolites in patients with hypertrophic cardiomyopathy (HCM) and healthy controls (Ctrl). **b**, Venn diagram depicting the number of upregulated PE metabolites in TAC-operated mice (left) and patients with HCM (right); the 6 PE metabolites common to both datasets are listed. **c**, FPKM $\log_2$ of *Acsl4* in patients with hypertrophic cardiomyopathy (HCM) and healthy controls (Ctrl). ns, not significant (unpaired Student's *t*-test).

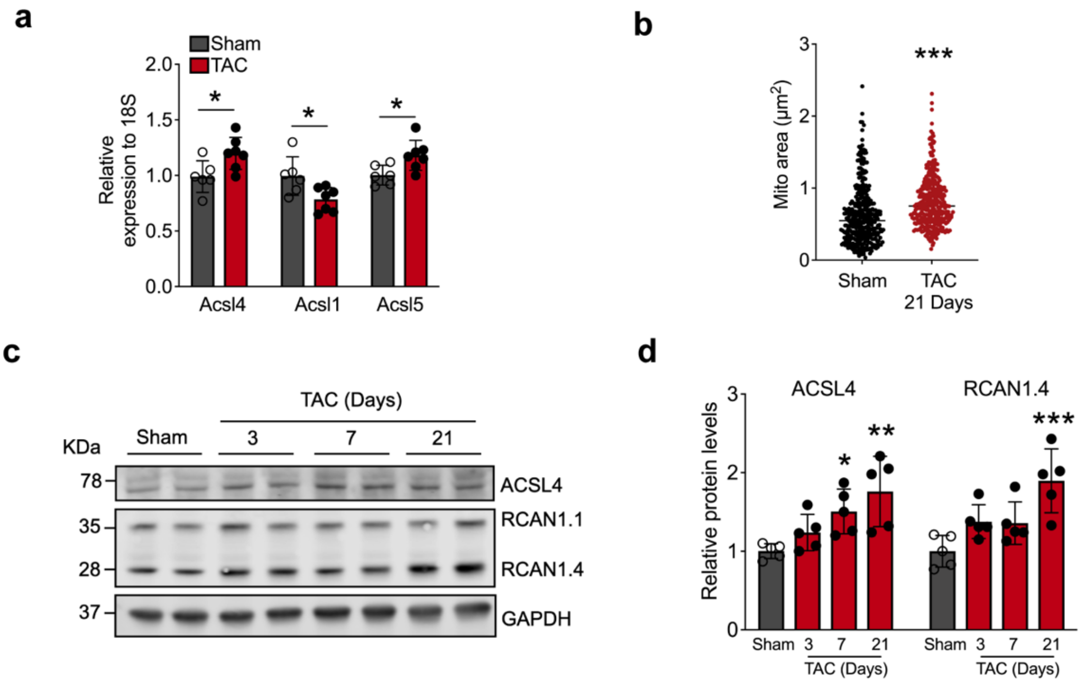

Supplementary Fig. 3 Acsl4 protein levels increase early following TAC. **a**, Summary of *Acsl4*, *Acsl1*, and *Acsl5* mRNA measured in the heart tissues of sham-operated and TAC-operated mice, 21 days after surgery, normalized to *18S* RNA and expressed relative to the respective sham-operated groups (n=6 mice/group). **b**, Corresponding relative mitochondrial area measured in left ventricular tissue obtained from sham-operated and TAC-operated mice (n=333-357 mitochondria from 5 mice/group); scale bars, 2 μm. **c** and **d**, Western blot analysis (c) and quantification (d) of ACSL4 and RCAN1.4 measured in the heart tissues of mice following sham surgery and 3, 7, and 21 days after TAC surgery (n=5 mice/group). *P<0.05, **P<0.01, and ***P<0.001 vs. the sham group (one-way ANOVA or unpaired Student's *t*-test).

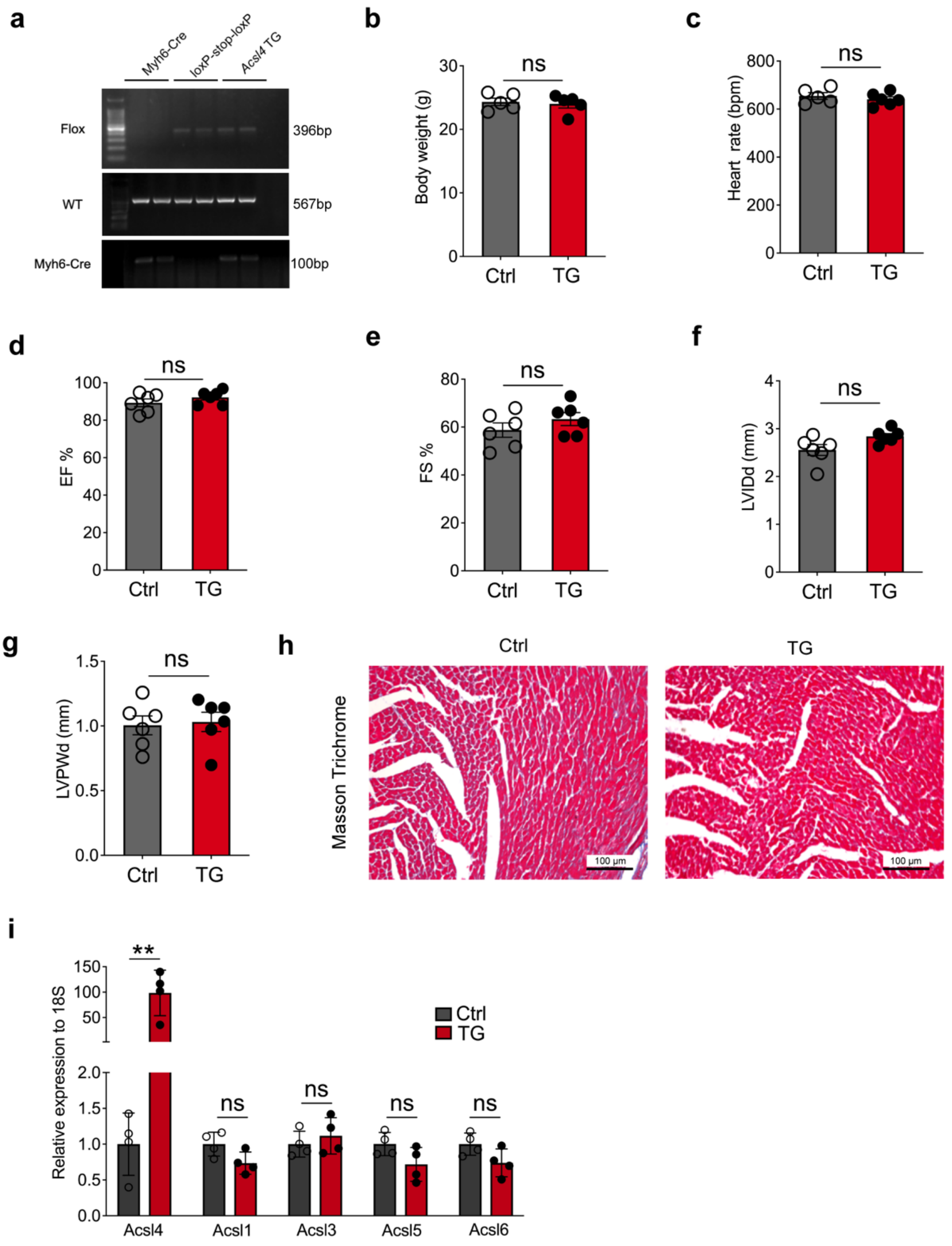

Supplementary Fig. 4 Characterization of cardiomyocyte-specific *Acsl4* transgenic mice. a, PCR analysis of genomic DNA to determine the presence or absence of the floxed allele, wild-type (WT) allele, and Myh6-Cre transgene in Myh6-Cre, *Acsl4*$^{flox/flox}$, and *Acsl4* TG mice. **b-g**, Summary of body weight (b), heart rate (c), ejection fraction (d), fractional shortening (e), left ventricular end-

diastolic internal dimension (f), and left ventricular posterior wall thickness at diastole (g) measured in Ctrl and *Acsl4* TG mice (n=5-6 mice/group). **h**, Example images of left ventricular tissue sections obtained from Ctrl and *Acsl4* TG mice and stained with Masson's trichrome (scale bar, 100 μm). **i**, Summary of *Acsl1* and *Acsl3* through *Acsl6* mRNA measured in the heart tissues of Ctrl and *Acsl4* TG mice, normalized to *18S* RNA and expressed relative to the respective Ctrl groups (n=4 mice/group). Note the break in *y*-axis. ns, not significant (unpaired Student's *t*-test).

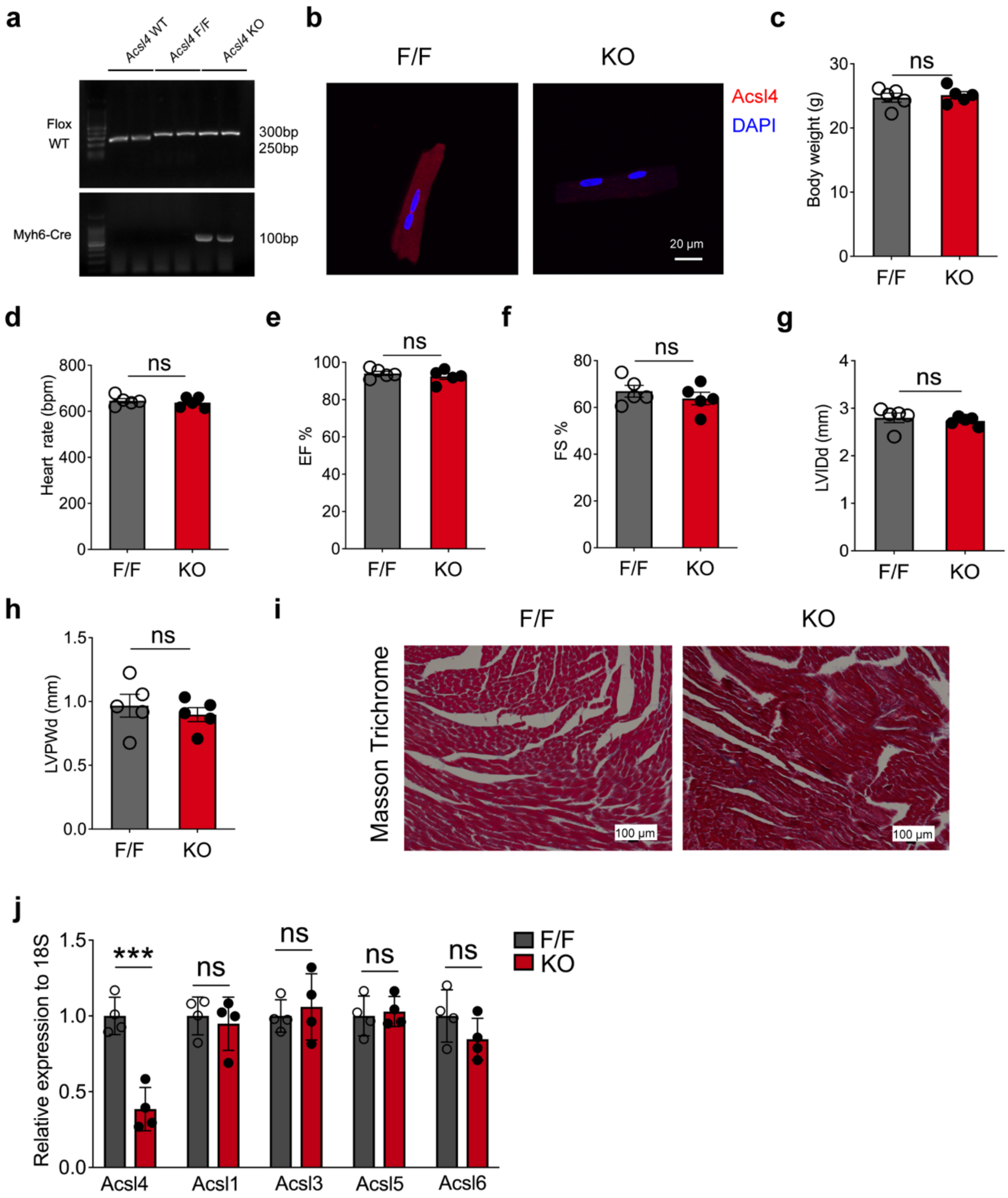

Supplementary Fig. 5 Characterization of cardiomyocyte-specific *Acsl4* knockout mice. a, PCR analysis of genomic DNA for the presence of the floxed allele, WT allele, and Myh6-Cre transgene in WT, *Acsl4*$^{flox/flox}$, and *Acsl4* knockout (KO) mice. **b**, Acsl4 immunofluorescence images of adult mouse ventricular myocytes obtained from adult control (F/F) and *Acsl4* KO mice; the nuclei were counterstained with DAPI (scale bar, 20 μm). **c-h**, Summary of body

weight (c), heart rate (d), ejection fraction (e), fractional shortening (f), left ventricular end-diastolic internal dimension (g), and left ventricular posterior wall thickness at diastole (h) measured in control (F/F) and *Acsl4* KO mice (n=5 mice/group). **i**, Example images of left ventricular tissue sections obtained from F/F and KO mice and stained with Masson's trichrome (scale bar, 100 μm). **j**, Summary of *Acsl1* and *Acsl3* through *Acsl6* mRNA measured in the heart tissues of control (F/F) and *Acsl4* KO mice, normalized to *18S* RNA and expressed relative to the respective F/F groups (n=4 mice/group). *** $P<0.001$ and ns, not significant (unpaired Student's *t*-test).

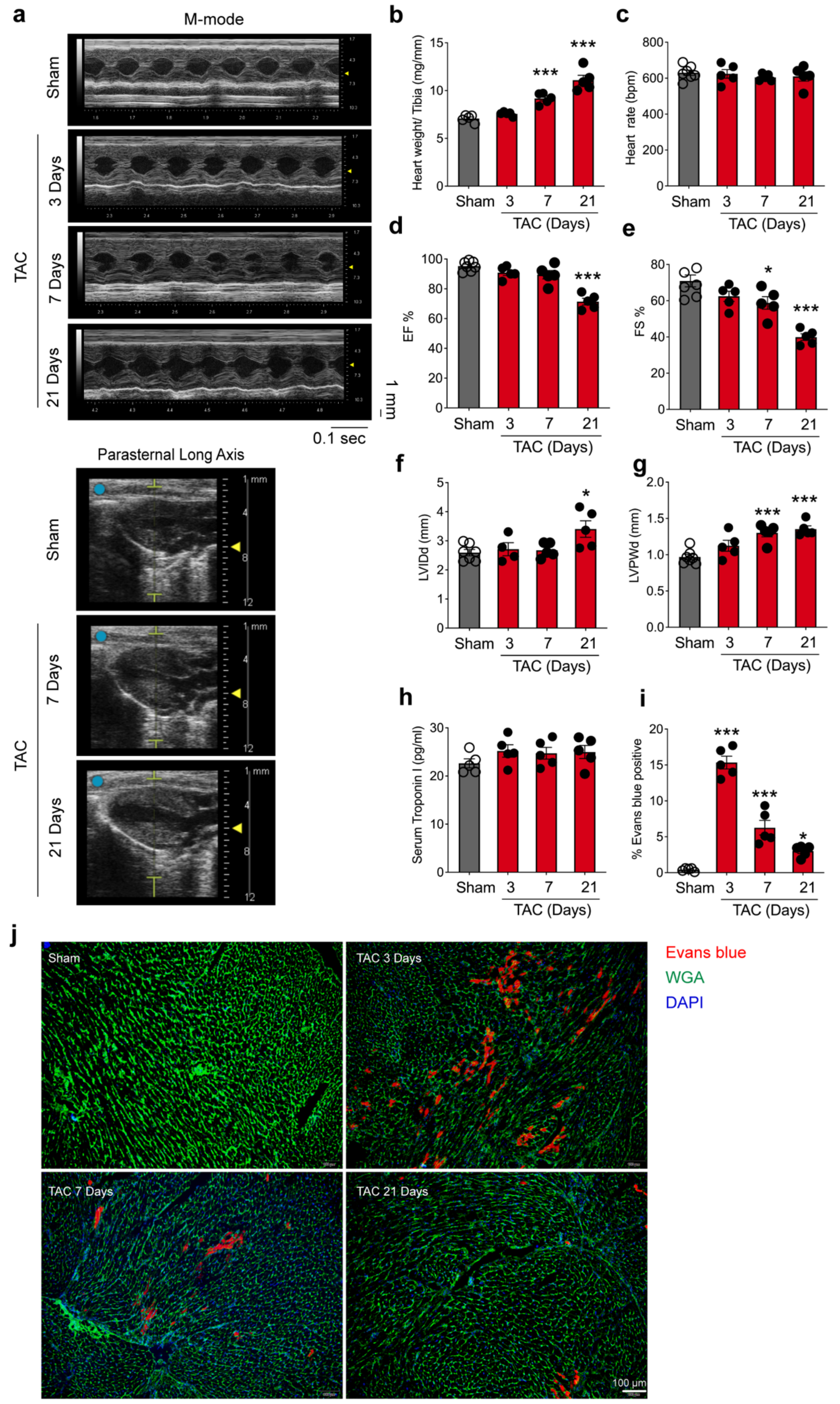
a
M-mode
Sham
TAC
3 Days
7 Days
21 Days
1 mm
0.1 sec
Parasternal Long Axis
Sham
TAC
7 Days
21 Days
b
Heart weight/ Tibia (mg/mm)
Sham 3 7 21
TAC (Days)
c
Heart rate (bpm)
Sham 3 7 21
TAC (Days)
d
EF %
Sham 3 7 21
TAC (Days)
e
FS %
Sham 3 7 21
TAC (Days)
f
LVIDd (mm)
Sham 3 7 21
TAC (Days)
g
LVPWd (mm)
Sham 3 7 21
TAC (Days)
h
Serum Troponin I (pg/ml)
Sham 3 7 21
TAC (Days)
i
% Evans blue positive
Sham 3 7 21
TAC (Days)
j
Sham
TAC 3 Days
TAC 7 Days
TAC 21 Days
100 μm
Evans blue
WGA
DAPI

Supplementary Fig. 6 Pressure overload induces cardiac dysfunction and cardiomyocyte death. a, Representative echocardiography images measured in WT mice following sham surgery or 3, 7, and 21 days after TAC surgery. **b-h**, Summary of the heart weight/tibia length ratio (b), heart rate (c), ejection fraction (d), fractional shortening (e), left ventricular end-diastolic internal dimension (f), left ventricular posterior wall thickness at diastole (g), and serum Troponin I levels (h) measured after sham surgery and 3, 7, and 21 days after TAC surgery (n=5 mice/group). **i** and **j**, Left ventricular tissues were obtained after sham surgery and 3, 7, and 21 days after TAC surgery and stained with wheat germ agglutinin (WGA, green). Evans blue–positive cardiomyocytes are shown in red, and the nuclei were counterstained with DAPI (blue). Shown are example fluorescence images (j) and the summary of Evans blue–positive cells (i) in the indicated mice (n=5 mice/group). Scale bar, 100 μm. *$P<0.05$ and ***$P<0.001$ vs. the sham group (one-way ANOVA).

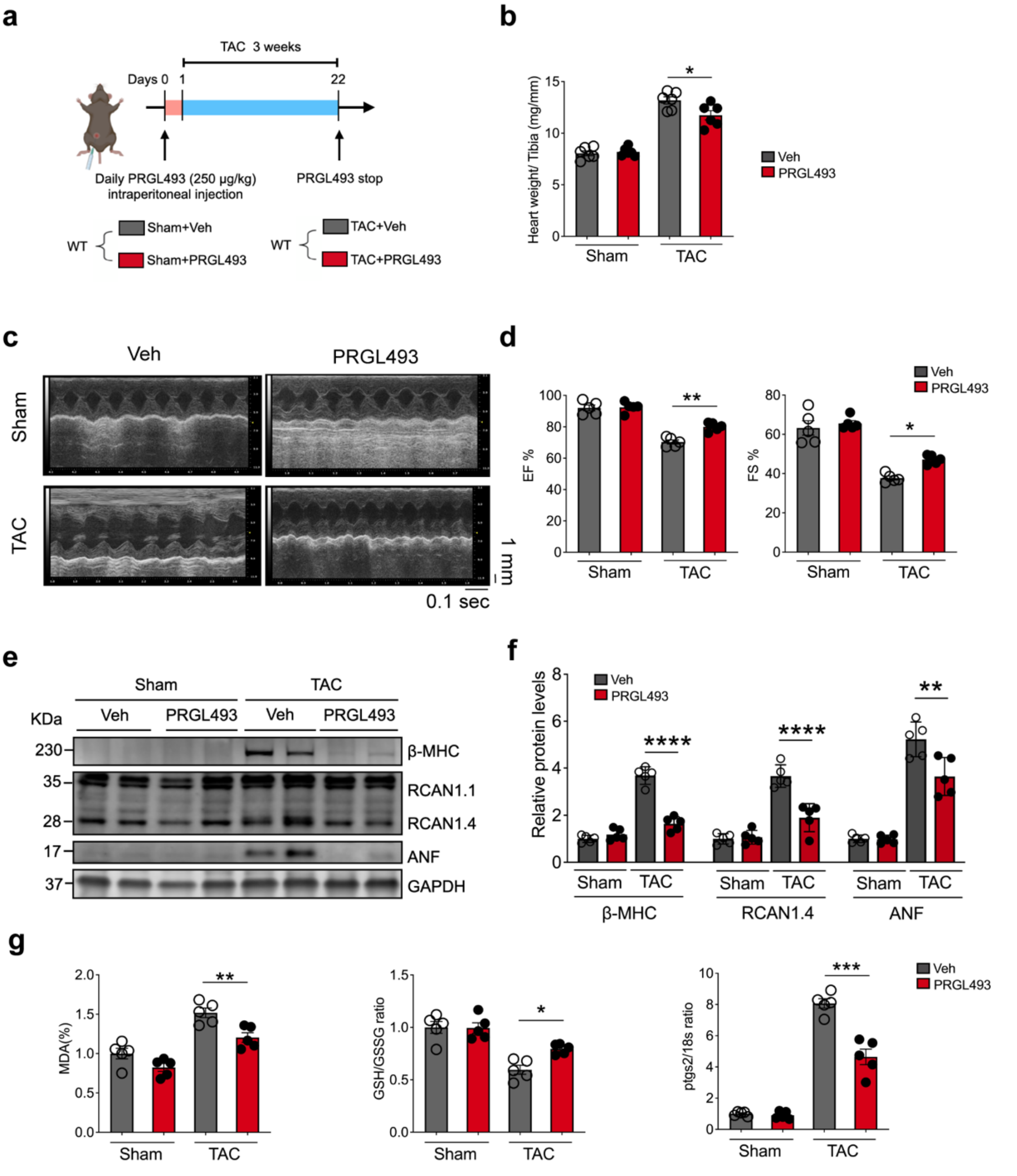

Supplementary Fig. 7 The Acsl4 inhibitor PRGL493 protects against impaired cardiac function in TAC-induced heart failure. **a**, Diagram depicting the strategy for inhibiting Acsl4 with daily i.p. injections of PRGL493 (or vehicle) starting on day 0; on day 1, the mice were subjected to sham surgery or TAC surgery, then analyzed 21 days later. **b**, Summary of the heart weight/tibia length ratio measured in vehicle-treated and PRGL493-treated

sham-operated and TAC-operated WT mice (n=6 mice/group). **c** and **d**, Example M-mode echocardiography images (c) and summary of ejection fraction (d, left) and fractional shortening (d, right) measured in the indicated groups (n=5 mice/group). **e** and **f**, Western blot analysis (e) and quantification (f) of the cardiac hypertrophy markers β-MHC, RCAN1.4, and ANF measured in the indicated groups, normalized to the respective sham-operated groups (n=5 mice/group). **g**, Summary of the change in ferroptosis markers MDA (malondialdehyde), GSH/GSSG (reduced glutathione/oxidized glutathione) ratio, and *Ptgs2* (prostaglandin-endoperoxide synthase 2) mRNA measured in the indicated groups, expressed relative to the respective vehicle-treated sham-operated groups (n=5 mice/group). **P*<0.05, ***P*<0.01 and ****P*<0.001 (two-way ANOVA).

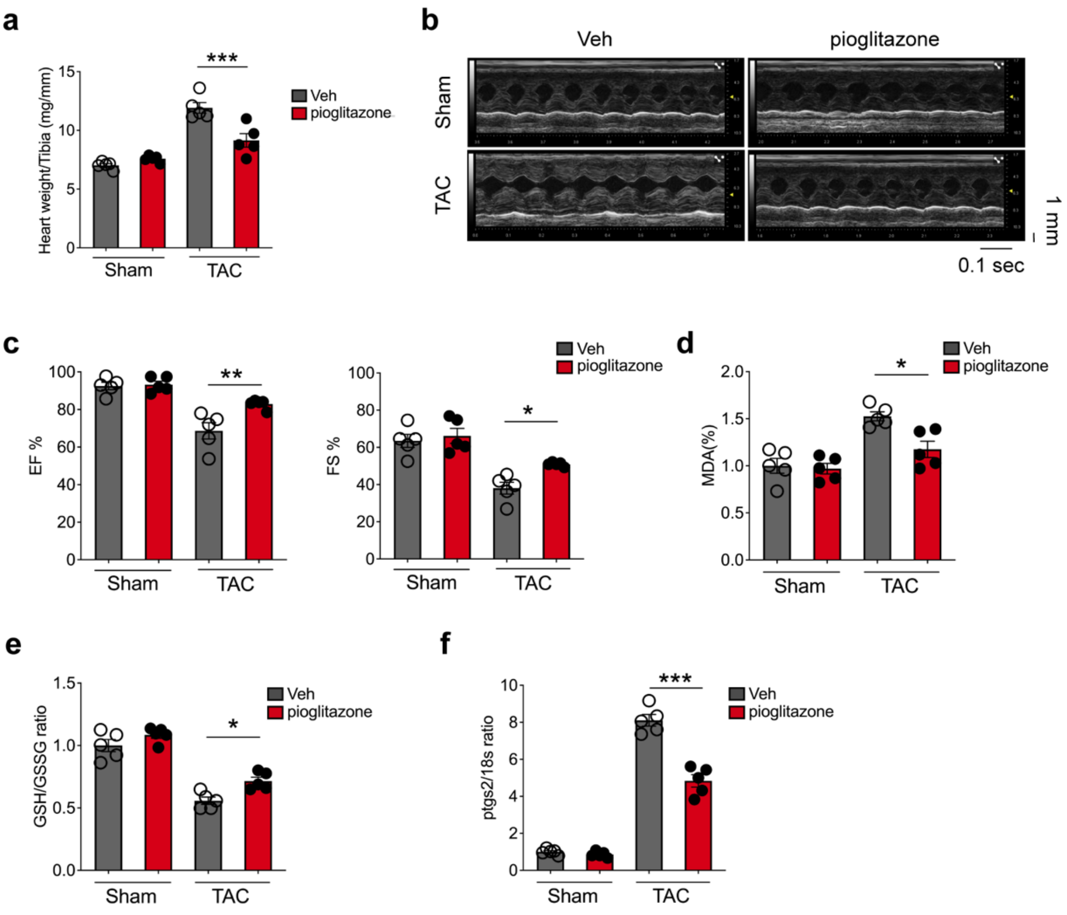

Supplementary Fig. 8 The Acsl4 inhibitor pioglitazone protects against impaired cardiac function in TAC-induced heart failure. a, Summary of the heart weight/tibia length ratio measured in vehicle-treated and pioglitazone-treated sham-operated and TAC-operated WT mice (n=5 mice/group). **b** and **c**, Example M-mode echocardiography images (b) and summary of ejection fraction (c, left) and fractional shortening (c, right) measured in the indicated groups (n=5 mice/group). **d-f**, Summary of change in MDA (d), the GSH/GSSG ratio (e), and *Ptgs2* mRNA (f) measured in the indicated groups, expressed relative to the respective vehicle-treated sham-operated groups (n=5 mice/group). **P*<0.05, ***P*<0.01 and ****P*<0.001 (two-way ANOVA).

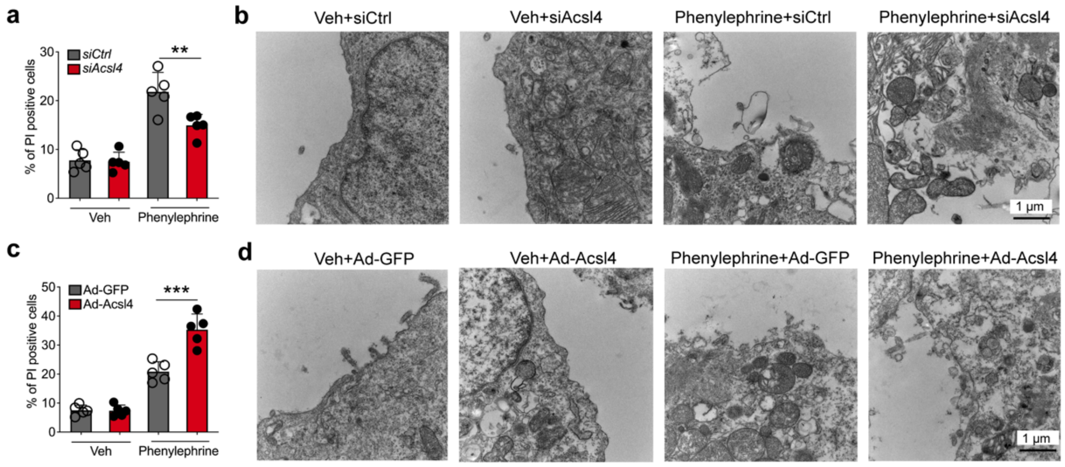

Supplementary Fig. 9 Hypertrophy stimuli induce cell death and membrane rupture of neonatal rat ventricular myocytes. a, Summary of the percentage of propidium iodide (PI)-positive NRVMs transfected with *siCtrl* or *siAcsl4* and then treated with vehicle (Veh) or phenylephrine (n=5). **b**, Representative transmission electron microscopy images of NRVMs transfected with *siCtrl* or *siAcsl4* and then treated with Veh or phenylephrine. **c**, Summary of the percentage of PI-positive NRVMs infected with an Acsl4-expressing adenovirus (Ad-Acsl4) or a GFP-expressing adenovirus (Ad-GFP) and then treated with vehicle or phenylephrine. (n=5). **d**, Representative transmission electron microscopy images of NRVMs infected with Ad-Acsl4 or Ad-GFP and then treated with vehicle or phenylephrine; scale bars, 1 μm.

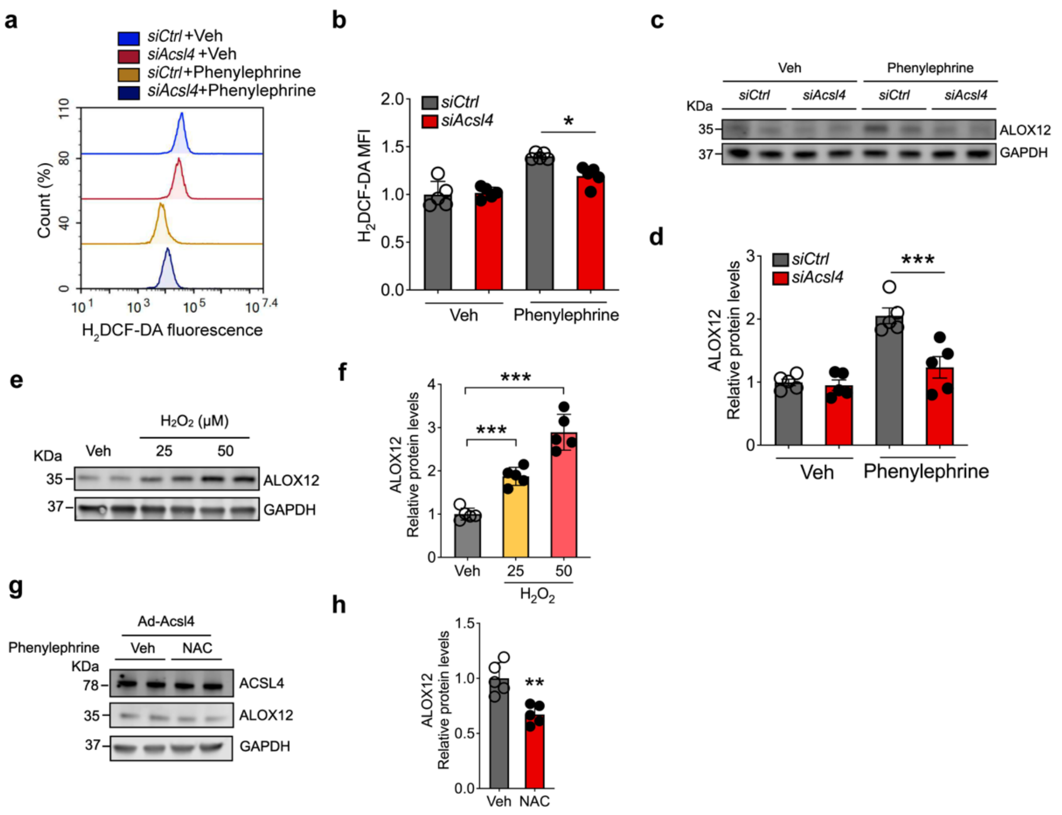

Supplementary Fig. 10 Acsl4-dependent ROS production upregulates Alox12 expression. a and **b**, Flow cytometry analysis (a) using H_2DCF-DA to measure ROS production, and quantification of H_2DCFDA mean fluorescence intensity (b) measured in NRVMs treated as indicated, normalized to the vehicle-treated *siCtrl* group (n=5 biological replicates/group). **c** and **d**, Western blot analysis (c) and quantification (d) of ALOX12 measured in NRVMs transfected with *siCtrl* or *siAcsl4* and then treated with vehicle or phenylephrine, normalized to the vehicle-treated *siCtrl* group (n=5 biological replicates/group). **e** and **f**, Western blot analysis (e) and quantification (f) of ALOX12 measured in NRVMs treated with vehicle, 25 μM H_2O_2, or 50 μM H_2O_2 for 24 hours, normalized to the vehicle-treated group (n=5 biological replicates/group). **g** and **h**, Western blot analysis (g) and quantification (h) of ALOX12 measured in NRVMs infected with Ad-Acsl4 and then treated with vehicle or the ROS scavenger *N*-acetyl-L-cysteine (NAC), normalized to the vehicle-treated group (n=5 biological replicates/group). *P<0.05, **P<0.01, and ***P<0.001 (two-way ANOVA, one-way ANOVA, or unpaired Student's *t*-test).

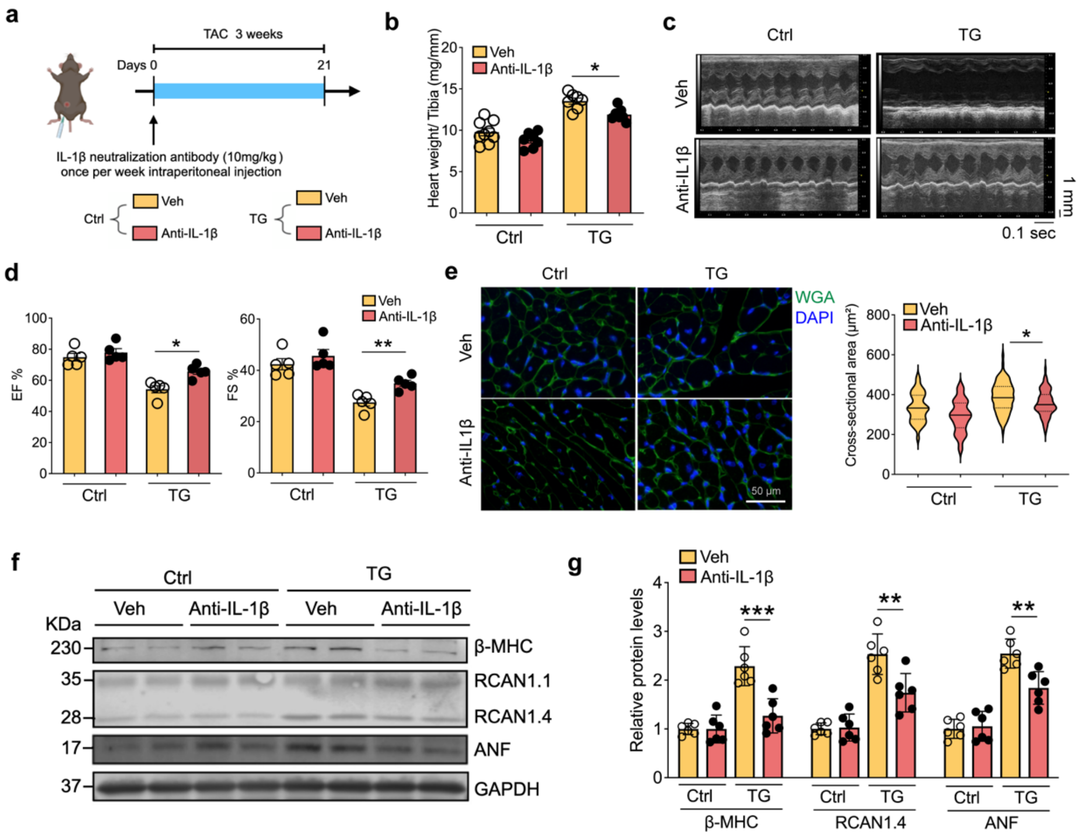

Supplementary Fig. 11 Increased IL-1β production triggered by Acsl4 promotes cardiac dysfunction. **a** and **b**, Diagram depicting the protocol for injecting mice with an IL-1β–neutralizing antibody (Anti-IL-1β) or vehicle (a), and summary (b) of the heart weight/tibia length ratio measured in vehicle-treated and anti-IL-1β–treated TAC-operated Ctrl and *Acsl4* TG mice (n=7-9 mice/group). **c** and **d**, M-mode echocardiography images (c) and summary of the ejection fraction (d, left) and fractional shortening (d, right) measured in the indicated TAC-operated mice (n=5 mice/group). **e**, Heart sections were prepared from the indicated TAC-operated mice and stained with WGA (green); the nuclei were counterstained with DAPI (blue). Scale bar, 50 μm. Shown at the right is a violin plot summarizing the relative cardiac cell cross-sectional area measured in the indicated mice (n=80-89 cardiomyocytes from 6 mice/group). **f** and **g**, Western blot analysis (f) and quantification (g) of the hypertrophy markers β-MHC, RCAN1.4, and ANF measured in heart tissues isolated from the indicated TAC-operated mice, normalized to the respective vehicle-treated control groups (n=6 mice/group). *P<0.05, **P<0.01, and ***P<0.001 (one-way or two-way ANOVA followed by Tukey's multiple comparisons test).

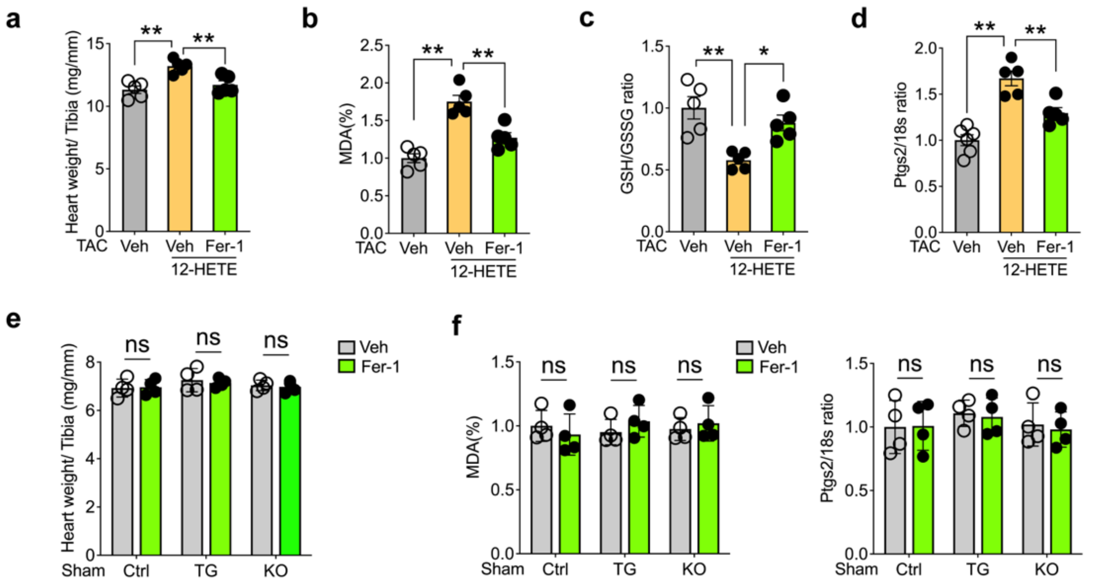

Supplementary Fig. 12 Acsl4-dependent ferroptosis induces pyroptotic signaling in response to TAC-induced pressure overload.

a-d, Summary of the heart weight/tibia length ratio (a), relative change in MDA levels (b), relative GSH/GSSG ratio (c), and relative *Ptgs2* mRNA normalized to *18S* (d) measured in TAC-operated WT mice treated as indicated (n=5 mice/group). **e-f**, Summary of the heart weight/tibia length ratio (e), relative change in MDA levels (f, left), and relative *Ptgs2* mRNA normalized to *18S* (f, right) measured in Fer-1–treated and vehicle-treated sham-operated control, *Acsl4* TG, and *Acsl4* KO mice. **P*<0.05, ***P*<0.01, and ns, not significant (two-way ANOVA, one-way ANOVA).

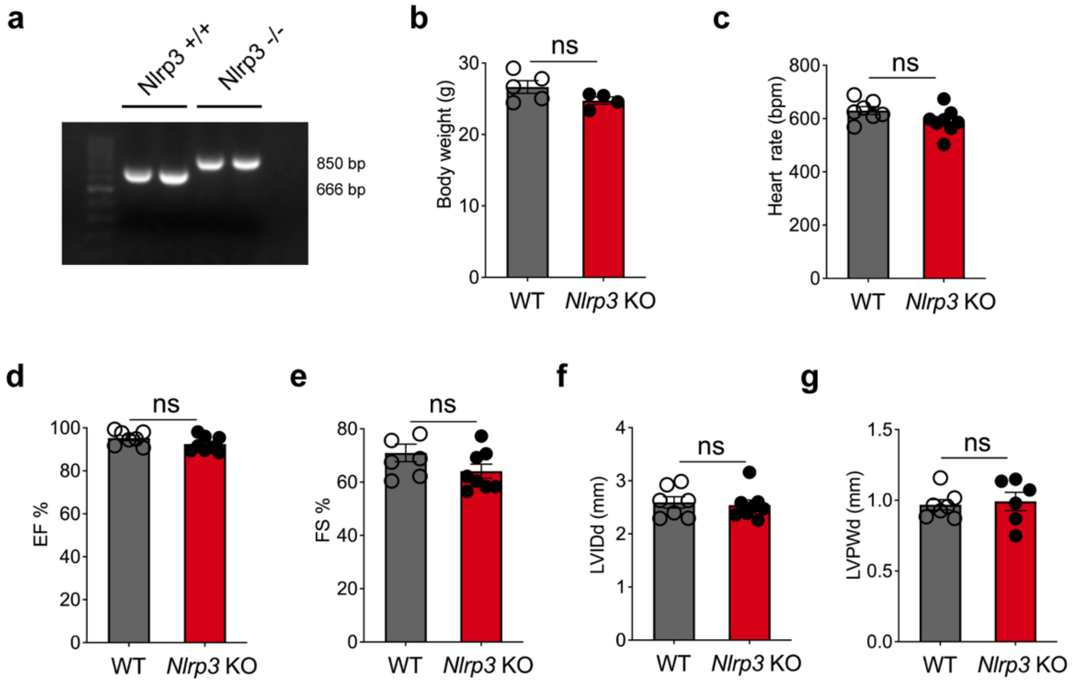

Supplementary Fig. 13 Characterization of global Nlrp3 knockout mice.
a, PCR analysis of genomic DNA to detect the WT and knockout *Nlrp3* alleles. **b-g**, Summary of body weight (b), heart rate (c), ejection fraction (d), fractional shortening (e), left ventricular end-diastolic internal dimension (f), and left ventricular posterior wall thickness at diastole (g) in WT and *Nlrp3* KO mice (n=4-7 mice/group). ns, not significant (unpaired Student's *t*-test).

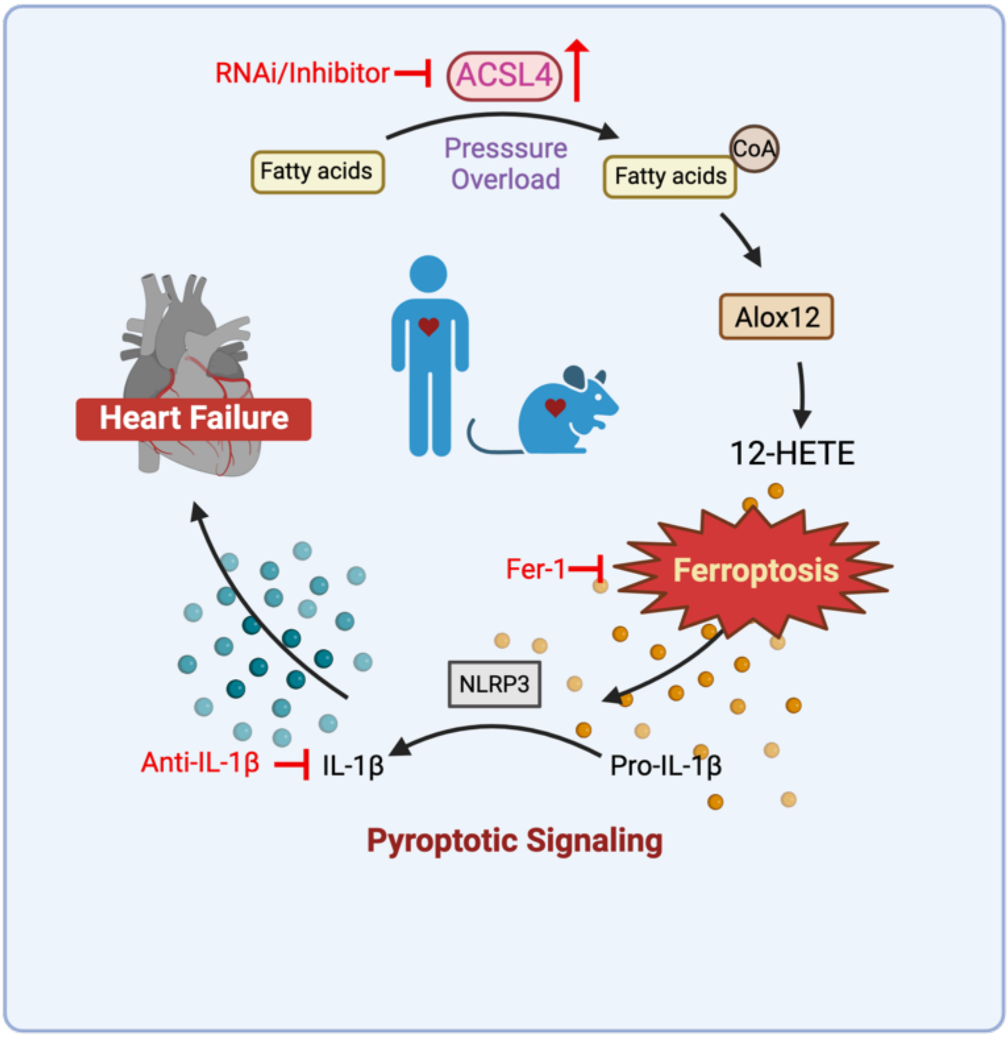

Supplementary Fig. 14 Proposed model depicting Acsl4-ferroptosis-triggered pyroptotic signaling in heart failure (Created with BioRender.com)**.** Upregulation of Acsl4 is identified as the key player in the murine model of cardiac hypertrophy. Acsl4 overexpression in cardiomyocytes promotes TAC-induced cardiac dysfunction and pathological remodeling in mice with heart failure by 12-HETE-mediated ferroptosis-triggered pyroptosis cascade. Either pharmacological inhibition or genetic deletion of Acsl4 significantly reduces left ventricular chamber hypertrophy and improves cardiac function.

Supplementary Table 1 Primer pairs used in this study for genotyping and qPCR.

Gene/allele	Species	Primer sequence 5' → 3'	Application
Acsl4 KO	mouse	AGTTAGCAGAGGGAGGCTGAATCT<br>ACCTCAGGATCTCAATCAAGTCAG	genotyping
Acsl4 TG	mouse	TTAATCCAGCCTGACTCCAACTA<br>CTGCCCTTACCTTACTACCACTGT	genotyping
Myh6-Cre	mouse	GATTTCGACCAGGTTCGTTC<br>GCTAACCAGCGTTTTCGTTC	genotyping
Nlrp3 KO	mouse	TGCCTGCTCTTTACTGAAGG<br>TTCCATTACAGTCACTCCAGATGT	genotyping
Nlrp3 WT	mouse	TCAGTTTCCTTGGCTACCAGA<br>TTCCATTACAGTCACTCCAGATGT	genotyping
Acsl1	mouse	CAGAACATGTGGGTGTCCAG<br>GTTACCAACATGGGCTGCTT	qPCR
Acsl3	mouse	GGGACTACAATACCGGCAGA<br>CCGCTGTCCATTTTCATCTT	qPCR
Acsl4	mouse	GCCAAAAGTGACCAGTCCTATG<br>TGTTACCAAACCAGTCTCGGGG	qPCR
Acsl5	mouse	AATGTGTTCAAAGGCTACCTAAAGGACCC<br>GCGACCAATGTCCCCAGTGTGA	qPCR
Acsl6	mouse	GCACAGAGCAGTTCGTTGGT<br>ATGTAGCTGATAGACCCTGGG	qPCR
Alox5	mouse	ACAGGGTCAAGAAGTTGGTGGG<br>CATCGTAGGAGTCCACCGC	qPCR
Alox12	mouse	AACCTAGTGCGTTTGTGGCT<br>CGTCGAAGTCAAACTCCTCC	qPCR
Alox15	mouse	AAGGTGATGAGCACAGGTGG<br>ATTCCCACCACGTACCGATT	qPCR
Ptgs2	mouse	CTGCGCCTTTTCAAGGATGG<br>GGGGATACACCTCTCCACCA	qPCR
18S	mouse	AGGGTTCGATTCCGGAGAGG<br>CAACTTTAATATACGCTATTGG	qPCR
