# Supplementary material for: Characterization of ferroptosis-triggered pyroptotic signaling in heart failure
Source: Signal Transduct Target Ther. 2024 Sep 25;9:257. doi: 10.1038/s41392-024-01962-6 (PMC11427671; doi:10.1038/s41392-024-01962-6)
Supplement: Supplementary file 2 — ORIGINAL GEL [file 41392_2024_1962_MOESM2_ESM.pdf]

Fig.1g

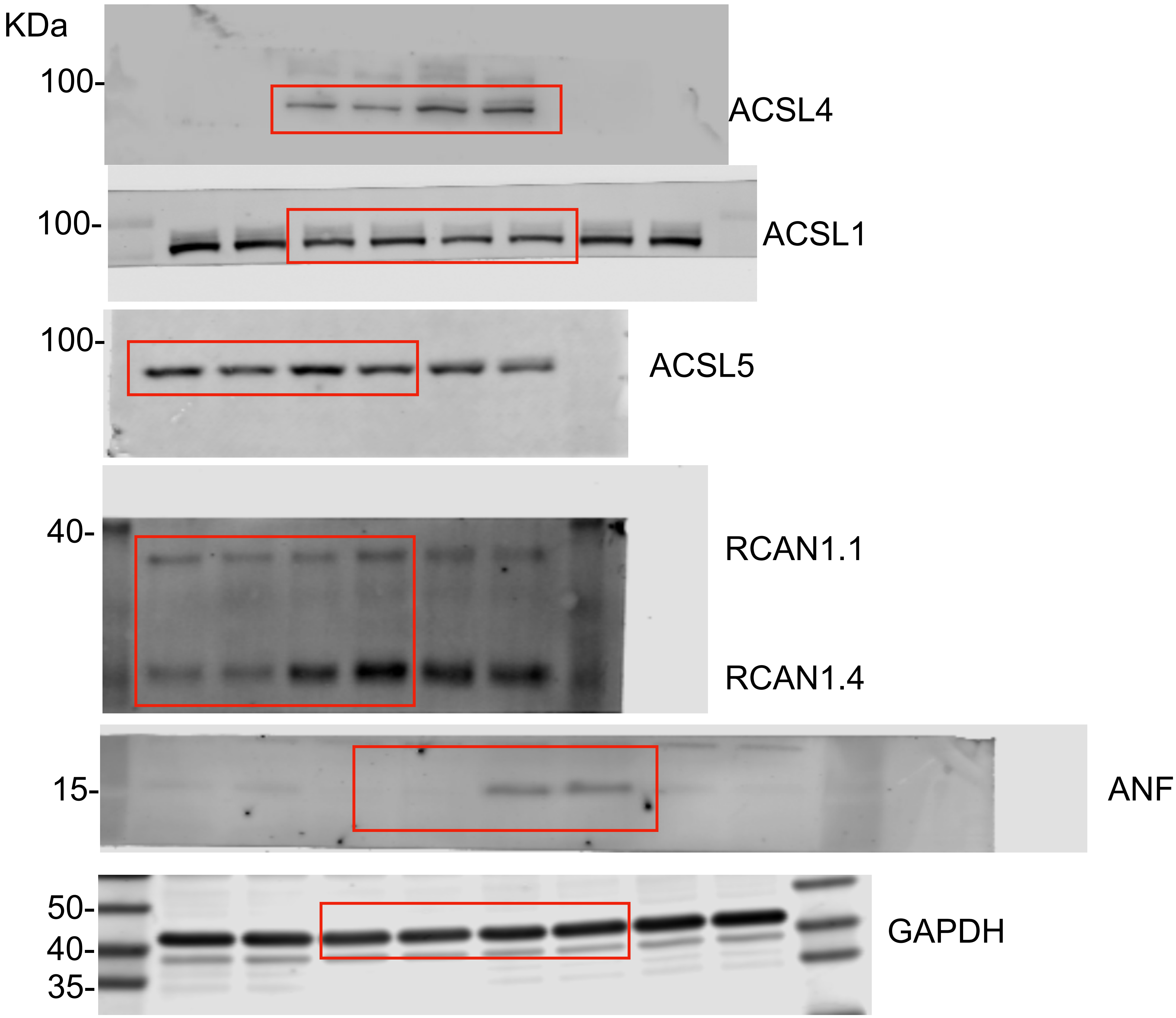
KDa
100-
ACSL4
100-
ACSL1
100-
ACSL5
40-
RCAN1.1
RCAN1.4
15-
ANF
50-
40-
35-
GAPDH

Fig.2i

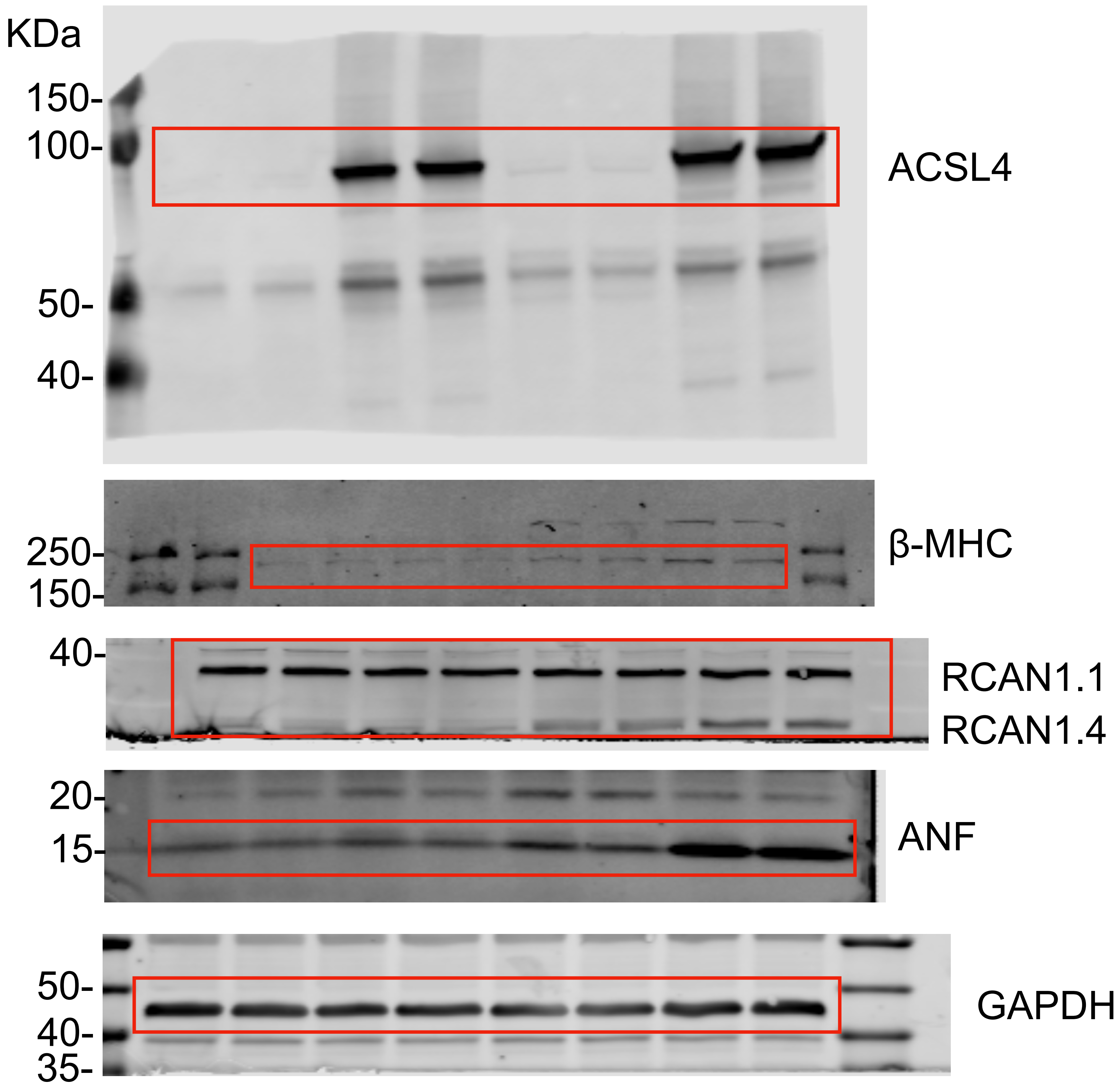
KDa
150-
100-
50-
40-
ACSL4
250-
150-
β-MHC
40-
RCAN1.1
RCAN1.4
20-
15-
ANF
50-
40-
35-
GAPDH

Fig.3i

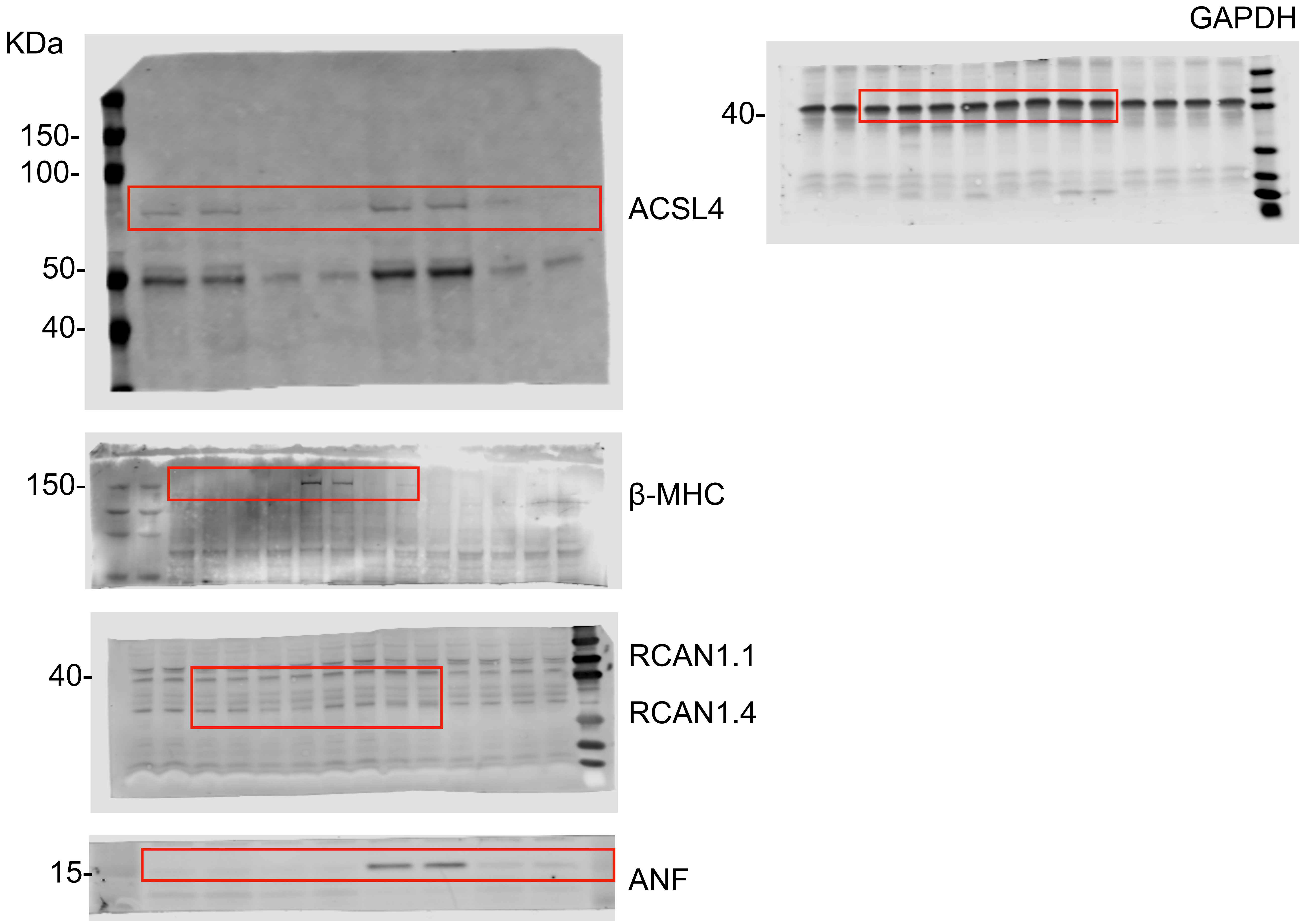
KDa
150-
100-
50-
40-
ACSL4
150-
β-MHC
40-
RCAN1.1
RCAN1.4
15-
ANF
GAPDH
40-

Fig.4a

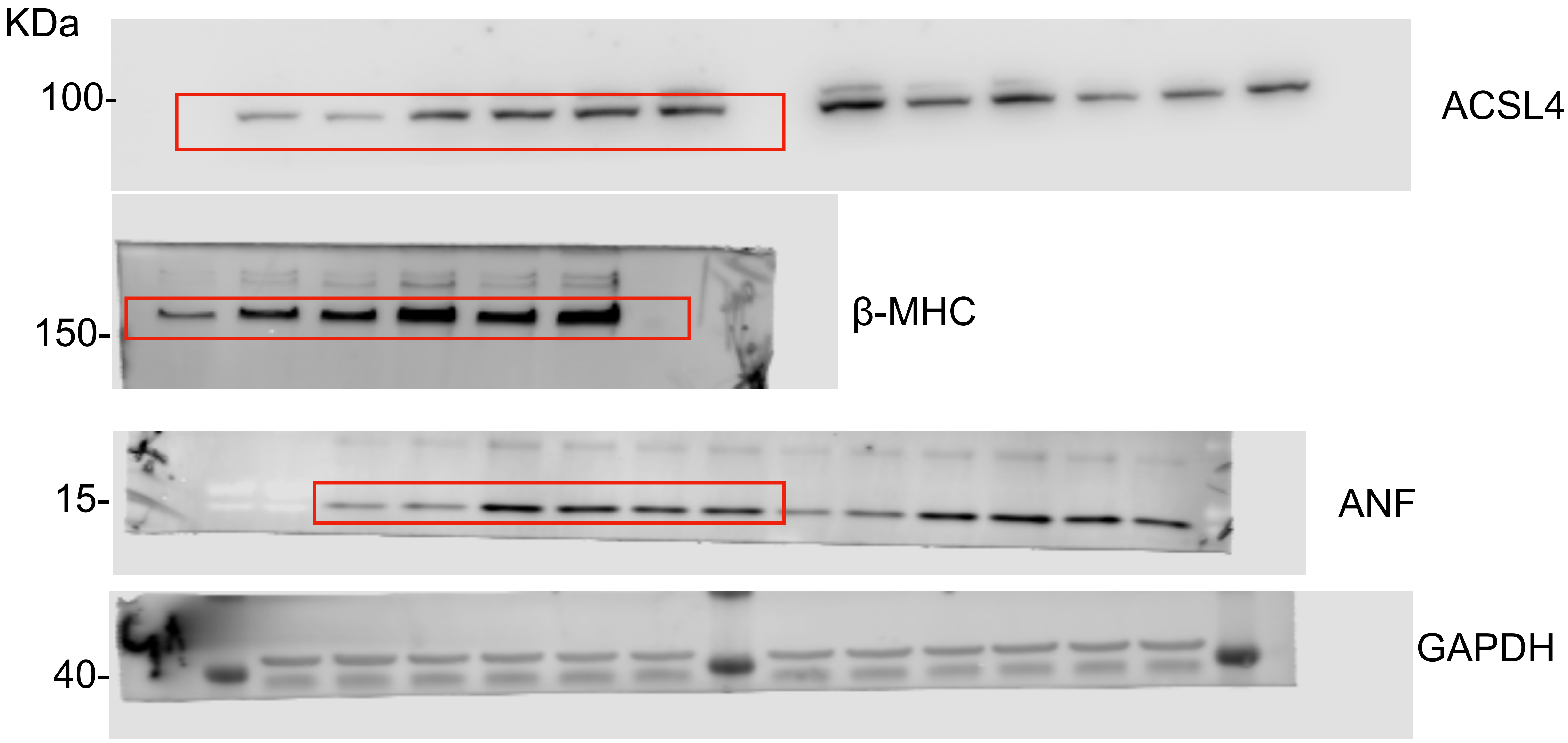

Fig.4d

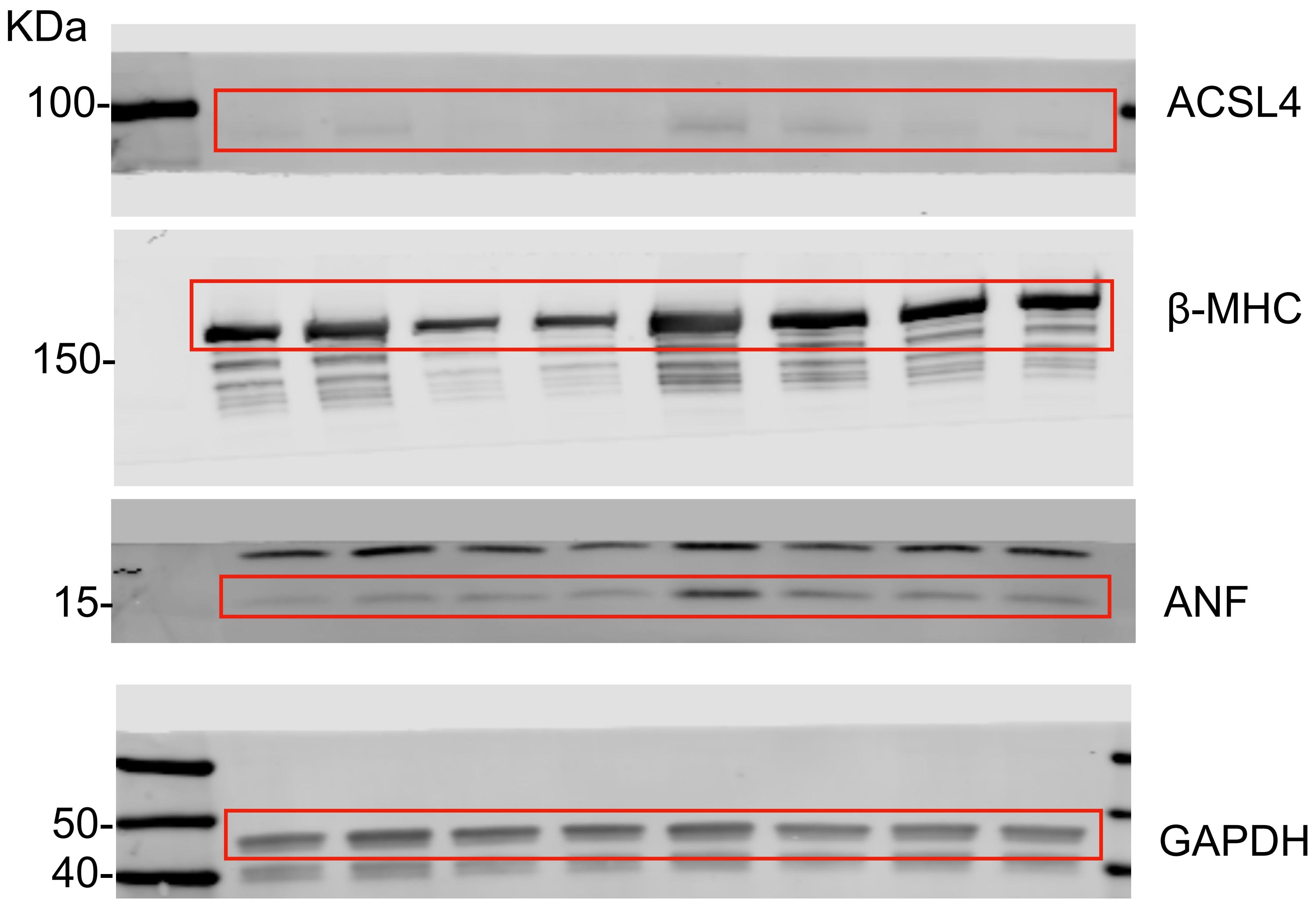

Fig.5h

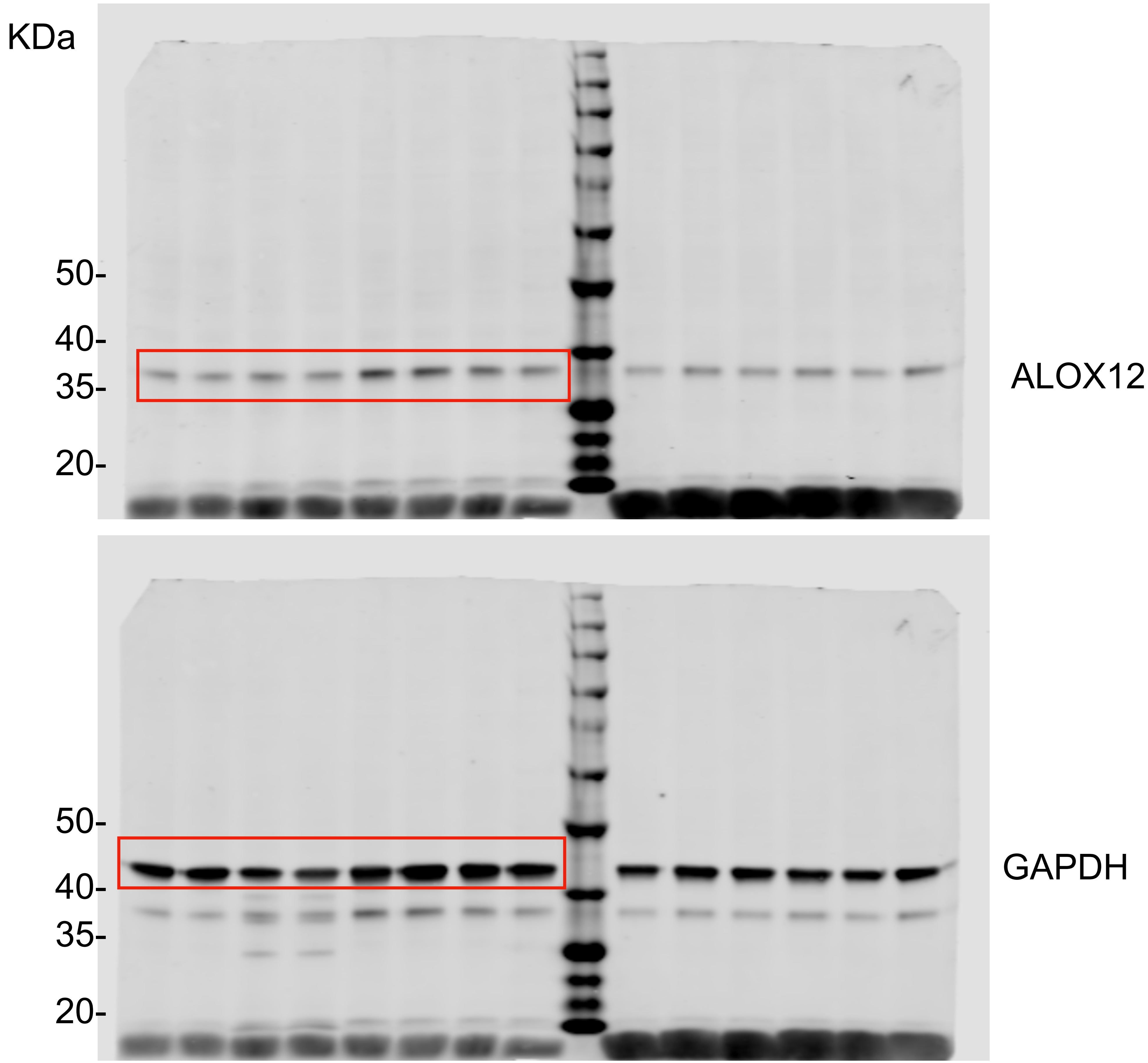

Fig.6e

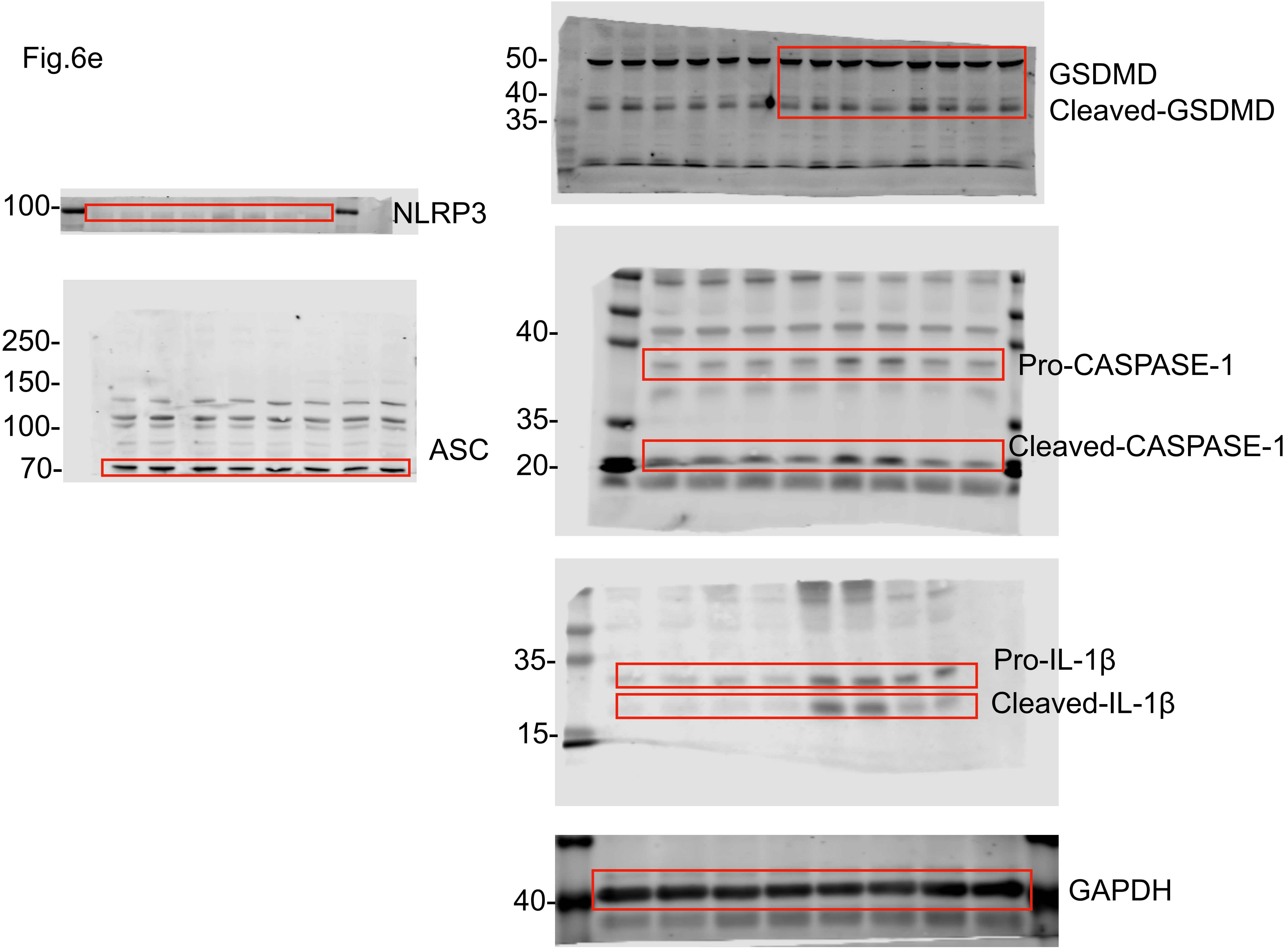
50-
40-
35-
GSDMD
Cleaved-GSDMD
100-
NLRP3
250-
150-
100-
70-
ASC
40-
Pro-CASPASE-1
35-
Cleaved-CASPASE-1
20-
35-
Pro-IL-1β
Cleaved-IL-1β
15-
40-
GAPDH

Fig.6g

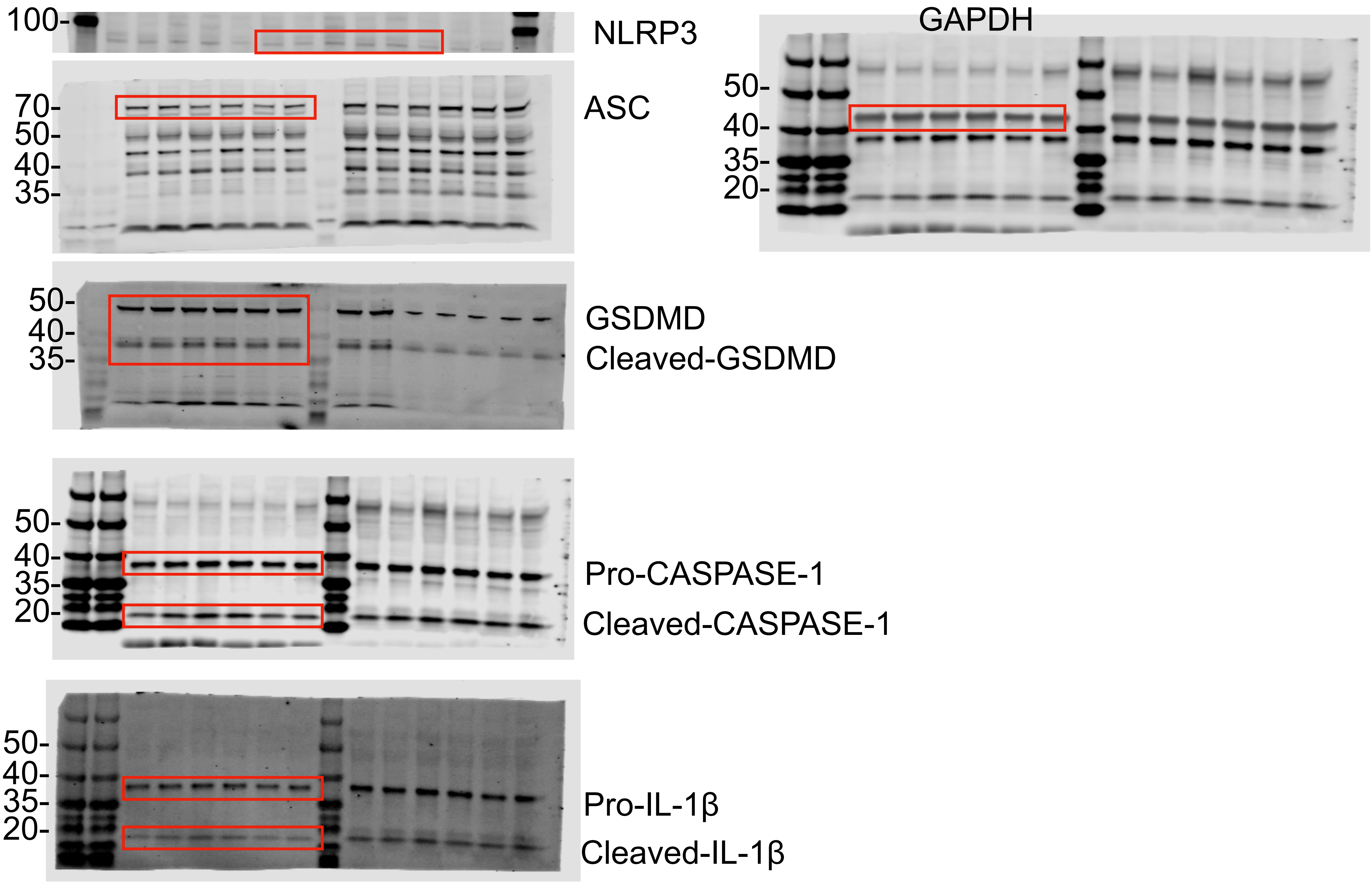
100-
NLRP3
70-
50-
40-
35-
ASC
50-
40-
35-
GSDMD
Cleaved-GSDMD
50-
40-
35-
20-
Pro-CASPASE-1
Cleaved-CASPASE-1
50-
40-
35-
20-
Pro-IL-1β
Cleaved-IL-1β
GAPDH
50-
40-
35-
20-

Fig.S3d

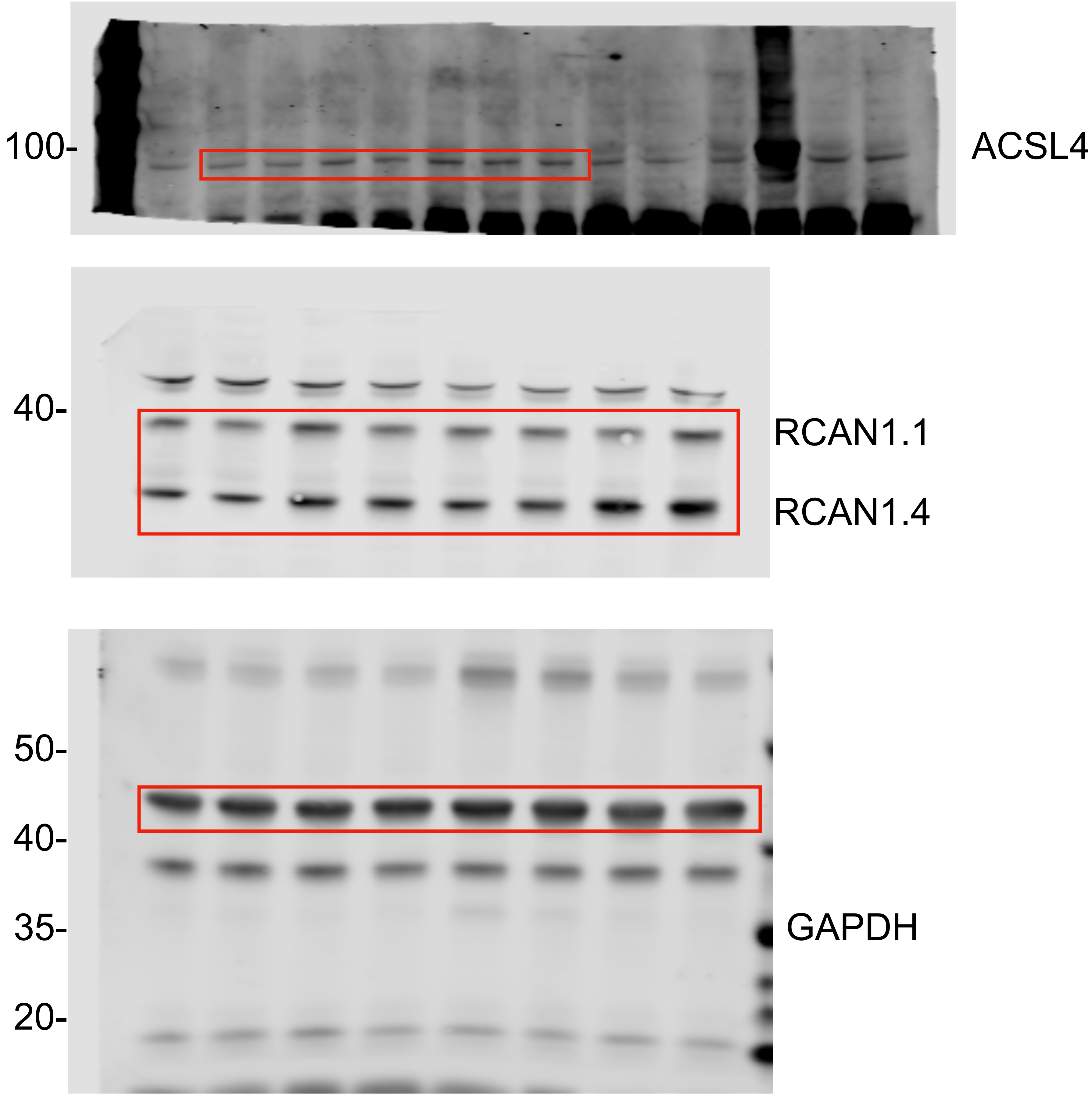

Fig.S7c

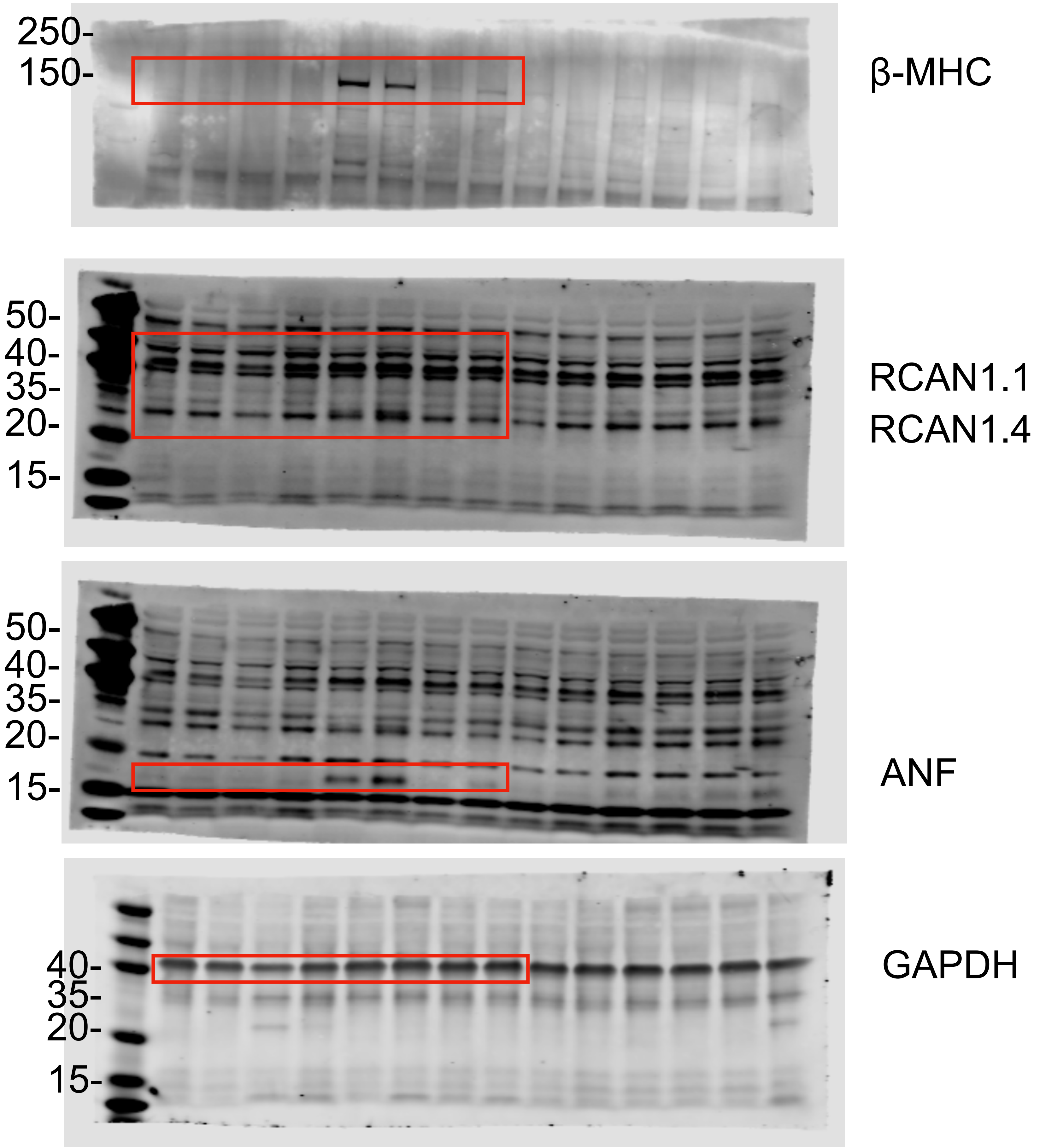
250-
150-
β-MHC
50-
40-
35-
20-
15-
RCAN1.1
RCAN1.4
50-
40-
35-
20-
15-
ANF
40-
35-
20-
15-
GAPDH

Fig.S10c

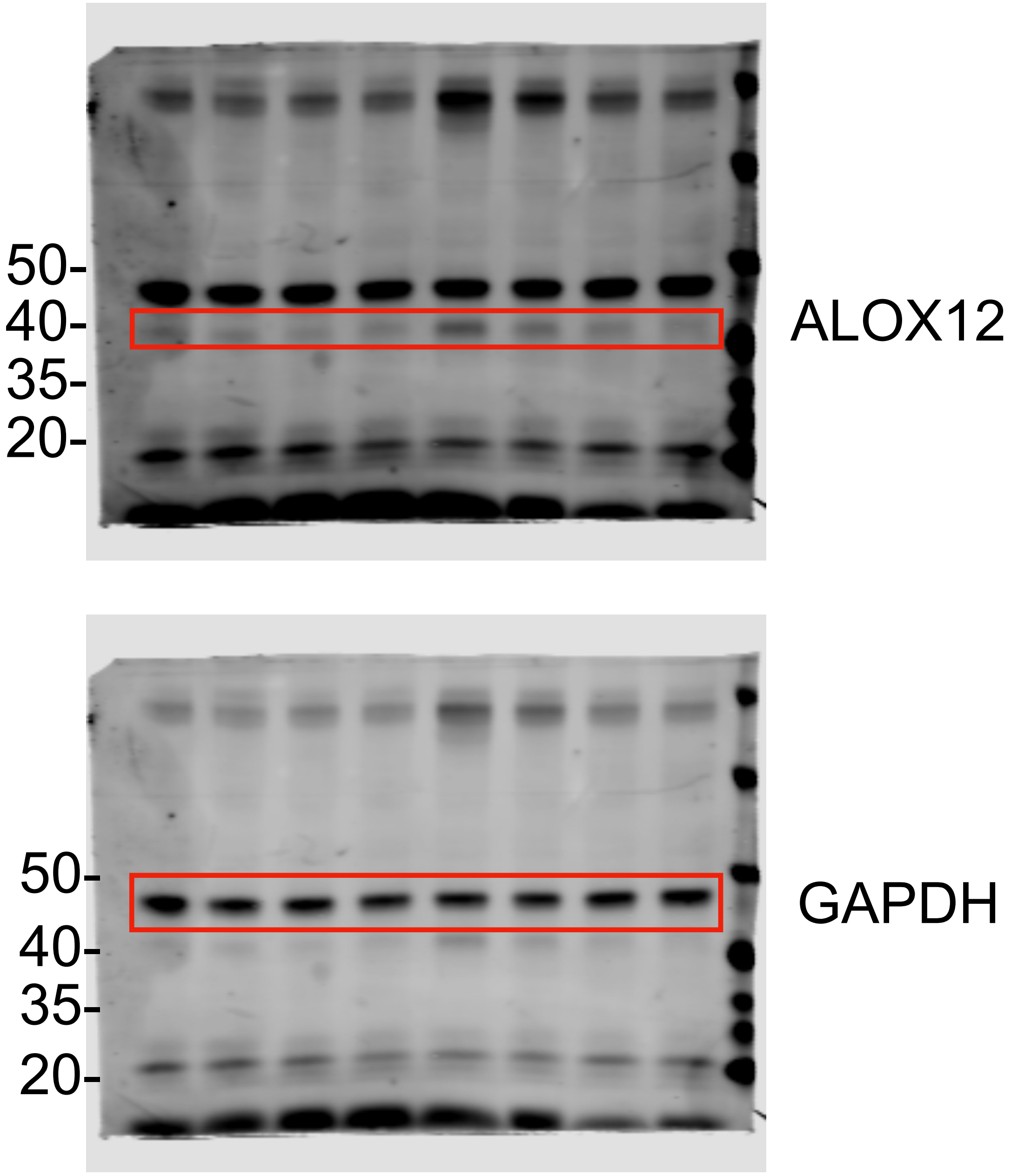

Fig.S10e

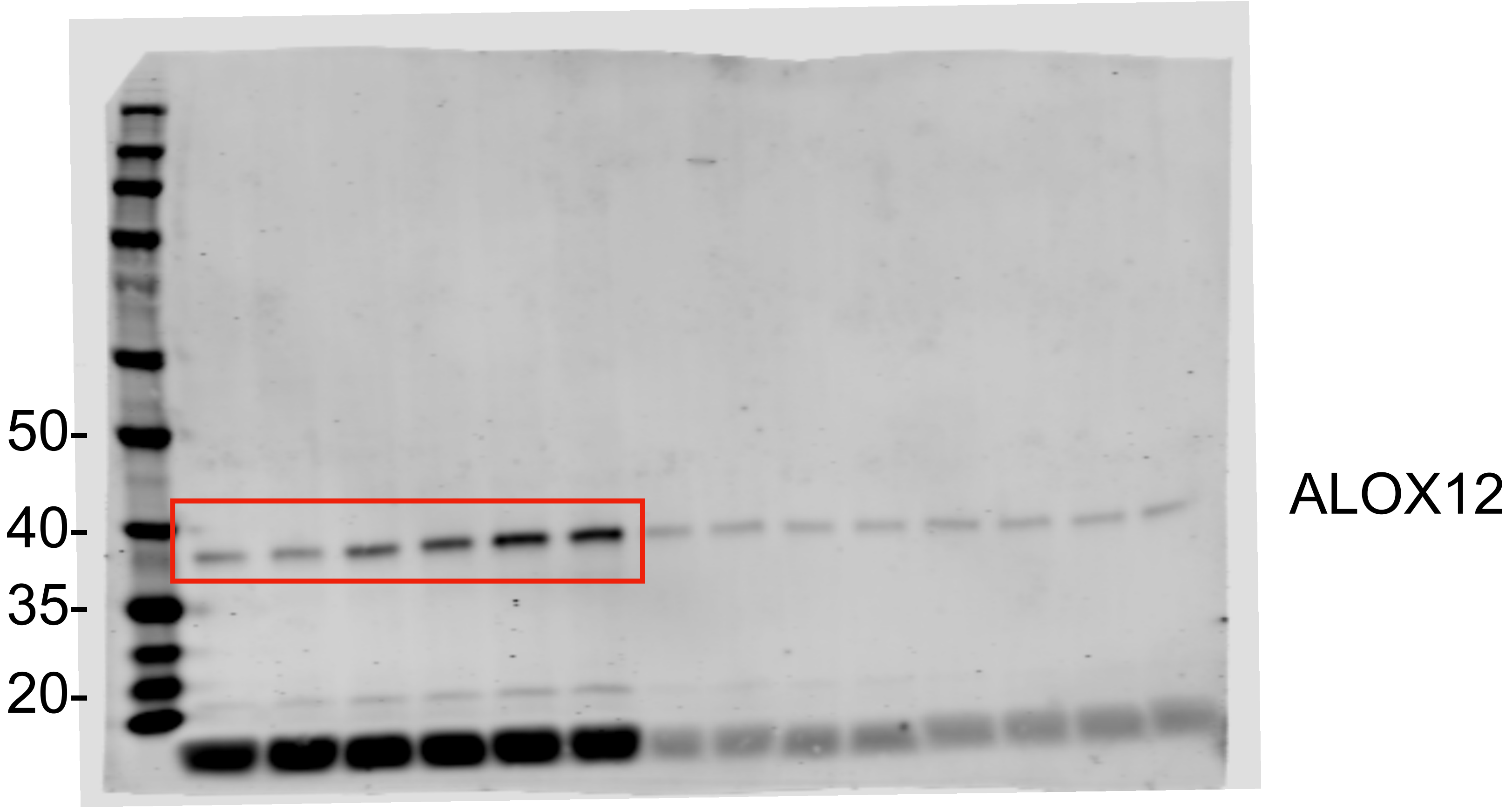

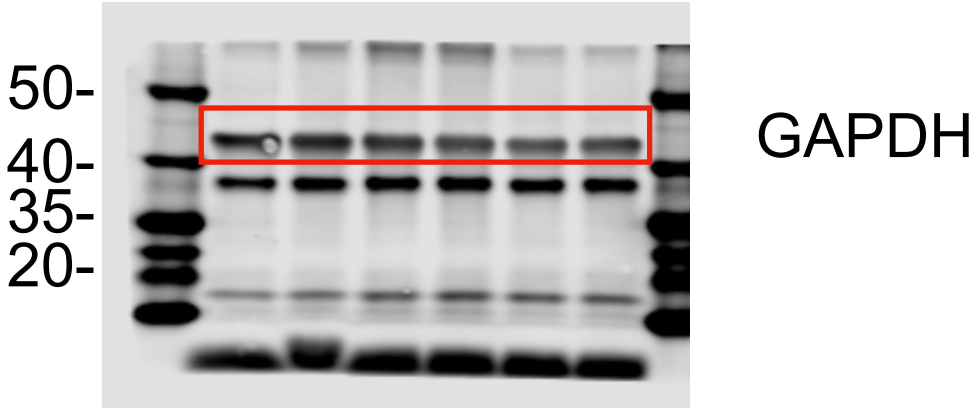

Fig.S10g

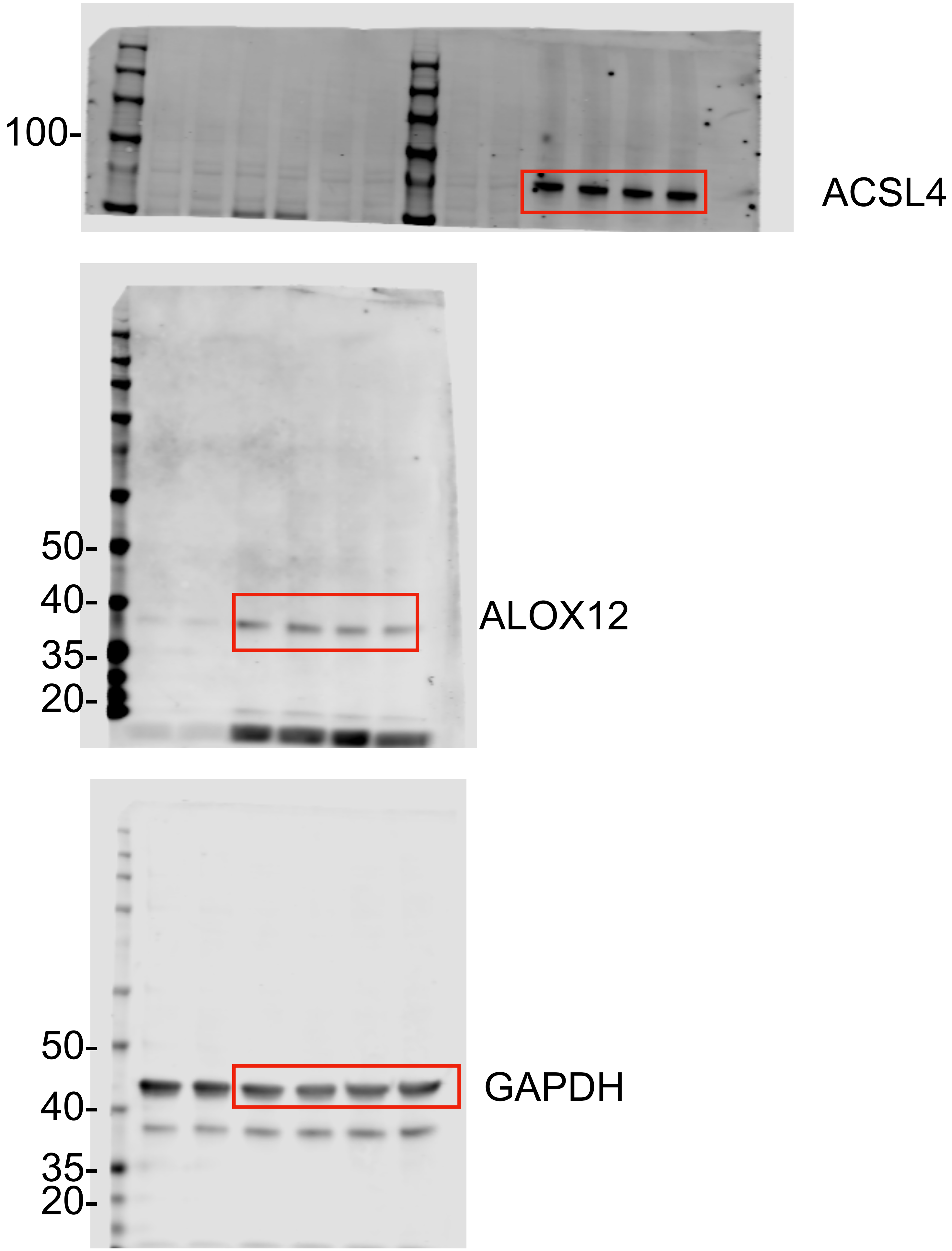

Fig.S11

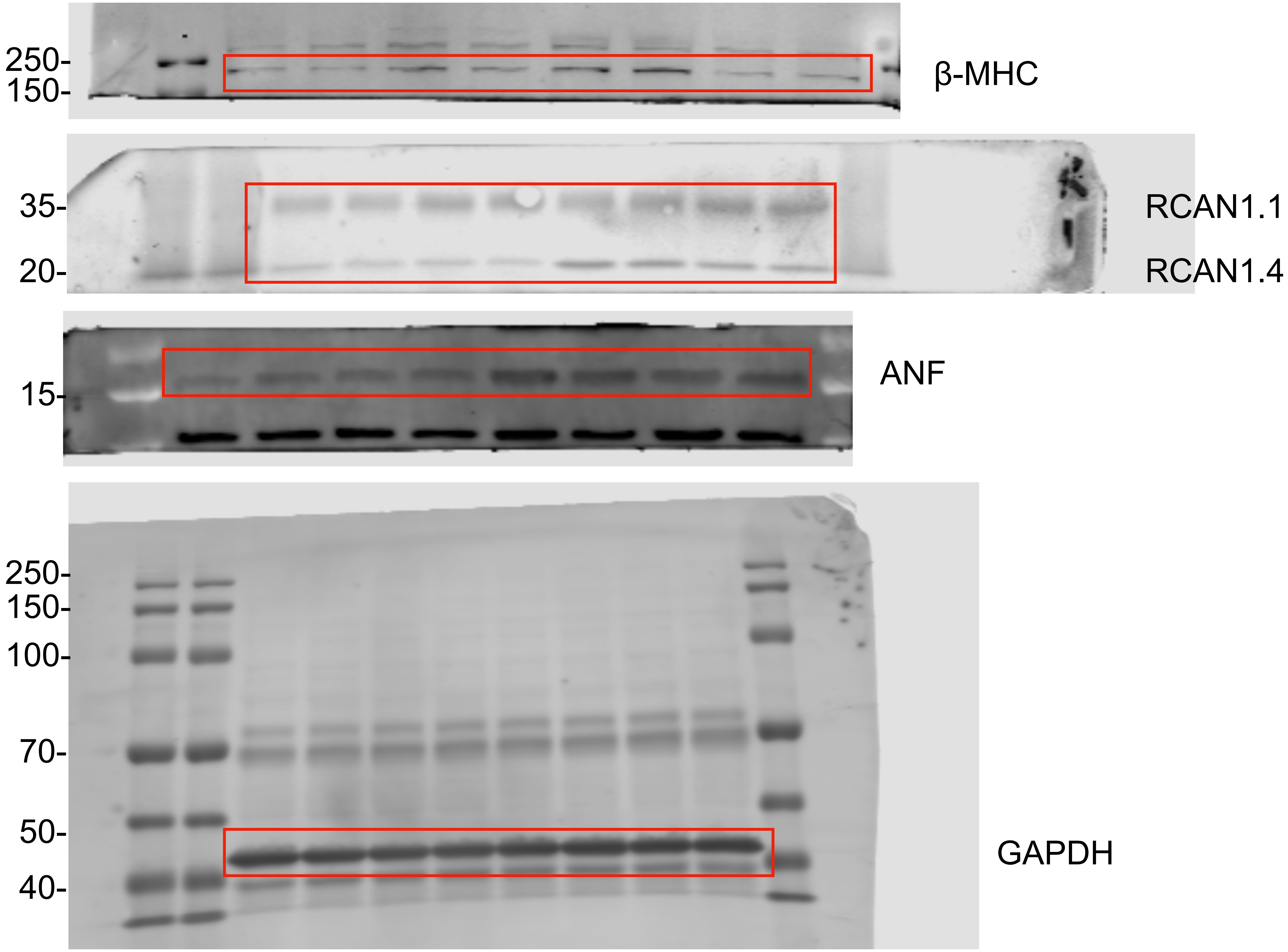
250-
150-
β-MHC
35-
20-
RCAN1.1
RCAN1.4
15-
ANF
250-
150-
100-
70-
50-
40-
GAPDH
